# Gram-positive pathogenic bacteria induce a common early response in human monocytes

**DOI:** 10.1186/1471-2180-10-275

**Published:** 2010-11-02

**Authors:** Svetlin Tchatalbachev, Rohit Ghai, Hamid Hossain, Trinad Chakraborty

**Affiliations:** 1 Institute of Medical Microbiology, Justus Liebig University, Frankfurter Str. 107, 35392 Giessen, Germany; 2 Evolutionary Genomics Group, Departamento Producción Vegetal y Microbiología, Universidad Miguel Hernández, San Juan de Alicante, Spain

## Abstract

**Background:**

We infected freshly isolated human peripheral monocytes with live bacteria of three clinically important gram-positive bacterial species, *Staphylococcus aureus*, *Streptococcus pneumoniae *and *Listeria monocytogenes *and studied the ensuing early transcriptional response using expression microarrays. Thus the observed response was unbiased by signals originating from other helper and effector cells of the host and was not limited to induction by solitary bacterial constituents.

**Results:**

Activation of monocytes was demonstrated by the upregulation of chemokine rather than interleukin genes except for the prominent expression of interleukin 23, marking it as the early lead cytokine. This activation was accompanied by cytoskeleton rearrangement signals and a general anti-oxidative stress and anti-apoptotic reaction. Remarkably, the expression profiles also provide evidence that monocytes participate in the regulation of angiogenesis and endothelial function in response to these pathogens.

**Conclusion:**

Regardless of the invasion properties and survival mechanisms of the pathogens used, we found that the early response comprised of a consistent and common response. The common response was hallmarked by the upregulation of interleukin 23, a rather unexpected finding regarding *Listeria *infection, as this cytokine has been linked primarily to the control of extracellular bacterial dissemination.

## Background

The percentage of patients with severe infections caused by gram-positive bacteria has increased in recent years, accounting for almost half of the incidents of septicemia and severe systemic infections [1-5]. A number of recent publications have investigated the transcriptional response to killed or inactive gram-positive pathogens, or the contribution of gram-positive cell wall constituents such as peptidoglycan (PepG), lipopeptide (LP) and lipoteichoic acid (LTA) to the triggering of specific host defense responses [6-10]. Though such studies are crucial for identifying stimulus specific effects, they are unable to account for the immunomodulatory effects of live bacteria, which frequently employ multiple survival strategies in parallel. Viable pathogenic bacteria secrete active components in the intercellular space and in the invaded cells in order to modulate the cellular response. In order to track the early events of gram-positive induced immune activation, we examined the total transcriptional response of isolated peripheral human CD14+/CD11b+ monocytes, infected with the viable bacterial pathogens: *Listeria monocytogenes*, *Staphylococcus aureus *and *Streptococcus pneumoniae *(hereafter referred to as LM, SA and SP respectively). All three pathogens belong to the group of low GC content bacteria. SP and SA are leading pathogens in cases of gram-positive sepsis and LM is a cause of meningitis in immunocompromised patients and also sepsis in newborns.

We designed and established a protocol enabling the detection of pathological changes early in the onset of infections with gram positive pathogens, before usual clinical parameters are upregulated, in an easily accessible cellular sample material. For these purposes, we focused our experimental analysis of naïve monocytes, which are easier to work with in *ex vivo *conditions than granulocytes, even though they are represented in much lower numbers *in vivo *than the latter. Peripheral monocytes also are among the first members of the host immune system to encounter pathogens after injury and epithelial penetration. We limited the infection to a short interval of 1 hour in the attempt to mimic the *in vivo *early reaction of the cells after first encountering the pathogen but before the onset of clinically manifested inflammation. Using microarray analysis, we were able to detect the transcriptional upregulation or repression of a robust minimal set of genes in infected cells compared to untreated controls in the short interval of one hour. Despite donor specific gene variations and despite the different invasion strategies of the bacteria studied, we identified a common program of gene expression induced by all three bacterial pathogens. This program is characterized by the upregulation of a key cytokine - interleukin 23 (IL23).

## Results

### Global response pattern of peripheral monocytes to infection

**Figure 1:**
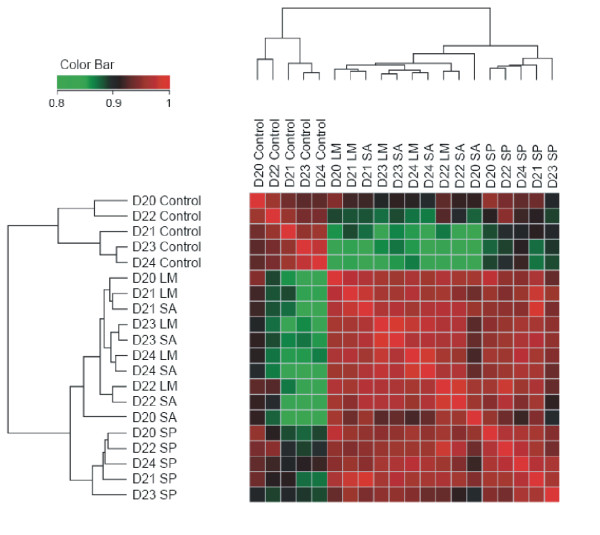
**Clustering of the correlation matrix of means for all microarray chips**. All arrays were compared to each other and the correlation between the expression values was determined. The matrix of correlation coefficients was clustered using hierarchical clustering with the euclidean distance metric. *L. monocytogenes *and *S. aureus *are clustered together, while controls and *S. pneumoniae *form separate clusters. D: Donor; Infection with: LM: *L. monocytogenes*, SA: *S. aureus*, SP: *S. pneumoniae*.

**Figure 2:**
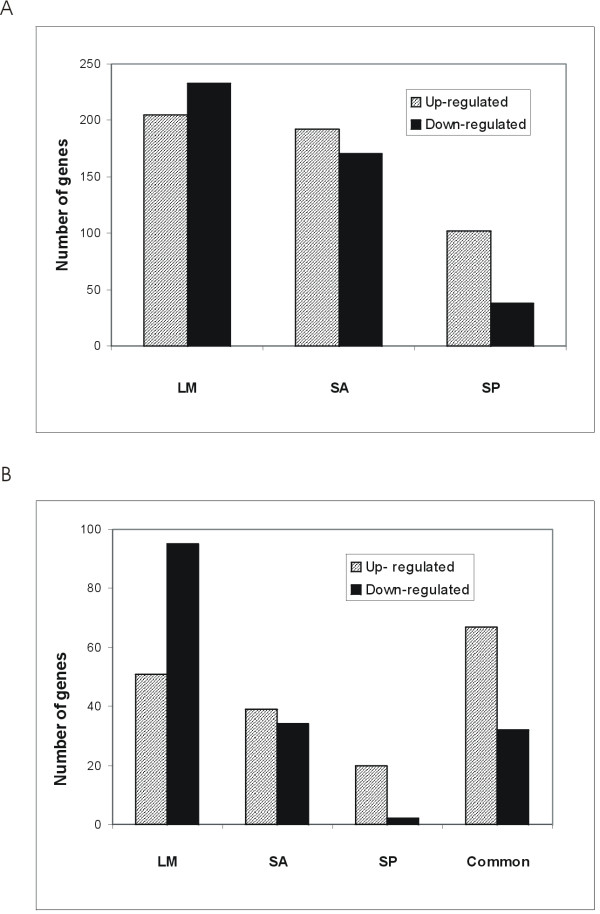
**Differentially expressed genes induced by each pathogen**. **(A)** Total upregulated and downregulated genes by each pathogen are represented as fold change values compared to the expression of the non-infected sample. (**B**) Comparison of specific and common induction of differentially expressed genes by each pathogen alone and by all three. *Listeria monocytogenes *induces the strongest common and specific gene regulation of all three pathogens fallowed by *S. aureus *and *S. pneumoniae*. LM: *L. monocytogenes *EGDe, SA: *S. aureus*, SP: *S. pneumoniae.*

To assess the global response we performed clustering of the correlation coefficients of the entire gene expression matrix comprising the unchallenged and the infected monocytes with all three pathogens (Figure 1). This revealed an interesting pattern. As can be seen from the figure, there are three main clusters. Cluster A comprising the controls, Cluster B comprising infection with *L. monocytogenes *(LM) and *S. aureus *(SA), and Cluster C comprising infection with *S. pneumoniae *(SP). We compared each pathogen to the control group to identify the differentially expressed genes (Figure 2). LM caused the induction of transcription of 205 and repression of 233 genes (Figure 2A; Additional files 1, 2, Tables S1, S2). The transcription of 192 genes was upregulated and 171 genes were downregulated upon infection with SA (Figure 2A; Additional files 3, 4, Tables S3, S4). For SP these numbers were smaller, with 102 and 38 genes upregulated respectively downregulated 1 h upon infection (Figure 2A; Additional files 5, 6, Tables S5, S6). Induction of target gene expression for the common upregulated genes was consistently higher for LM and SA than SP. All differentially expressed genes by pathogen with fold changes are available as additional files (Additional files 1, 2, 3, 4, 5, 6, Tables S1-S6).

### Common and pathogen specific responses of peripheral monocytes

All pathogens induced a common set of 66 upregulated and 32 downregulated genes (Tables 1, 2, Figure 2B). Consistent with common core responses against pathogenic stimuli [11], we observed genes involved in proinflammation, chemotaxis, suppression of immune response and adhesion molecules. LM induced the largest number of pathogen-specific transcription changes, especially downregulating 95 genes (Figure 2B; Additional files 7, 8, Tables S7, S8), compared with 34 by SA (Figure 2B; Additional files 9, 10, Tables S9, S10). Only two genes (out of a total of 38 downregulated) were individually downregulated by SP and 20 genes were upregulated only by infection with SP (Figure 2B; Additional files 11, 12, Tables S11, S12). All of the common regulated genes sorted by Gene Ontology (GO) are available as additional file (Additional file 13, Excel work sheet S1).

**Table 1:** List of commonly upregulated genes for all pathogens.

				Fold Change		
**No**.	**Gene IDs**	**Gene Symbol**	**Gene name**	**LM**	**SA**	**SP**

1	51561	IL23A	Interleukin 23, alpha subunit p19""	48,15	30,02	6,70

2	57124	CD164L1	CD248 antigen, endosialin""	20,36	22,03	8,23

3	3426	IF	I factor (complement)	17,07	13,08	6,88

4	10411	RAPGEF3	Rap guanine nucleotide exchange factor (GEF) 3	13,85	11,81	5,60

5	1960	EGR3	Early growth response 3	12,16	8,98	2,65

6	3725	JUN	V-jun sarcoma virus 17 oncogene homolog (avian)	10,80	12,09	6,35

7	9389	SLC22A14	Solute carrier family 22 (organic cation transporter), member 14""	10,41	9,29	5,40

8	6648	SOD2	Superoxide dismutase 2, mitochondrial""	10,24	8,61	4,23

9	3290	HSD11B1	Hydroxysteroid (11-beta) dehydrogenase 1	10,04	20,04	5,73

10	122616	C14orf79	Chromosome 14 open reading frame 79	9,27	8,15	4,45

11	4790	NFKB1	Nuclear factor of kappa light polypeptide gene enhancer in B-cells 1 (p105)	8,03	8,17	3,06

12	131566	DCBLD2	Discoidin, CUB and LCCL domain containing 2""	7,94	5,46	4,59

13	1906	EDN1	Endothelin 1	7,76	10,71	3,40

14	9025	RNF8	Ring finger protein 8	7,38	6,01	2,98

15	51458	RHCG	Rhesus blood group, C glycoprotein""	6,90	8,94	2,55
16	1735	DIO3	Deiodinase, iodothyronine, type III""	6,83	6,20	3,70
17	9021	SOCS3	Suppressor of cytokine signaling 3	6,82	9,90	3,10

18	83740	H2AFB3	H2A histone family, member B3""	6,69	8,58	3,55

19	9028	RHBDL1	Rhomboid, veinlet-like 1 (Drosophila)""	6,18	5,07	2,28

20	8659	ALDH4A1	Aldehyde dehydrogenase 4 family, member A1""	6,13	3,74	3,02

21	8740	TNFSF14	Tumor necrosis factor (ligand) superfamily, member 14""	5,74	3,83	1,60

22	51025	Magmas	Mitochondria-associated protein involved in granulocyte-macrophage colony-stimulating factor signal transduction	5,52	5,54	3,11

23	10120	ACTR1B	ARP1 actin-related protein 1 homolog B, centractin beta (yeast)""	5,17	4,63	2,70

24	2615	GARP	Leucine rich repeat containing 32	4,96	3,18	3,03

25	55655	NALP2	NACHT, leucine rich repeat and PYD containing 2""	4,72	4,59	2,83

26	6004	RGS16	Regulator of G-protein signalling 16	4,66	2,42	2,22

27	6935	TCF8	Transcription factor 8 (represses interleukin 2 expression)	4,66	2,81	7,11

28	3280	HES1	Hairy and enhancer of split 1, (Drosophila)""	4,65	4,37	2,02

29	983	CDC2	Cell division cycle 2, G1 to S and G2 to M""	4,64	4,21	2,31

30	9076	CLDN1	Claudin 1	4,60	4,85	2,59

31	10307	APBB3	Amyloid beta (A4) precursor protein-binding, family B, member 3""	4,53	3,47	2,90

32	283131	TncRNA	Trophoblast-derived noncoding RNA	4,29	5,03	2,53

33	114112	TXNRD3	Thioredoxin reductase 3	4,15	3,55	2,01

34	6355	CCL8	Chemokine (C-C motif) ligand 8	4,06	3,54	2,54

35	1164	CKS2	CDC28 protein kinase regulatory subunit 2	3,95	3,12	2,11

36	5142	PDE4B	Phosphodiesterase 4B, cAMP-specific (phosphodiesterase E4 dunce homolog, Drosophila)""	3,93	3,93	2,35

37	6358	CCL14	Chemokine (C-C motif) ligand 14	3,80	4,33	3,76

38	788	SLC25A20	Solute carrier family 25 (carnitine/acylcarnitine translocase), member 20""	3,72	3,69	2,55

39	3697	ITIH1	Inter-alpha (globulin) inhibitor H1	3,71	3,12	2,05

40	2322	FLT3	Fms-related tyrosine kinase 3	3,66	4,59	2,20

41	6489	SIAT8A	ST8 alpha-N-acetyl-neuraminide alpha-2,8-sialyltransferase 1""	3,47	3,23	2,67

42	23529	CLC	Cardiotrophin-like cytokine factor 1	3,33	7,64	2,12

43	55647	RAB20	RAB20, member RAS oncogene family""	3,31	3,86	3,95

44	54847	SIDT1	SID1 transmembrane family, member 1""	3,15	4,43	2,78

45	10514	MYBBP1A	MYB binding protein (P160) 1a	3,09	5,53	2,35

46	64108	IFRG28	28 kD interferon responsive protein	3,08	2,48	2,22

47	9590	AKAP12	A kinase (PRKA) anchor protein (gravin) 12	2,99	2,72	2,09

48	5894	RAF1	V-raf-1 murine leukemia viral oncogene homolog 1	2,97	2,75	2,19

49	51365	PLA1A	Phospholipase A1 member A	2,95	4,52	2,45

50	6696	SPP1	Secreted phosphoprotein 1 (osteopontin, bone sialoprotein I, early T-lymphocyte activation 1)""	2,93	5,05	1,70

51	1939	LGTN	Ligatin	2,80	3,90	2,15

52	57801	HES4	Hairy and enhancer of split 4 (Drosophila)	2,78	2,72	2,13

53	3202	HOXA5	Homeo box A5	2,76	2,07	2,37

54	4216	MAP3K4	Mitogen-activated protein kinase kinase kinase 4	2,68	3,54	2,05

55	481	ATP1B1	ATPase, Na+/K+ transporting, beta 1 polypeptide""	2,65	2,04	2,27

56	1466	CSRP2	Cysteine and glycine-rich protein 2	2,64	8,75	3,66

57	11182	SLC2A6	Solute carrier family 2 (facilitated glucose transporter), member 6""	2,48	2,46	2,00

58	83660	TLN2	Talin 2	2,41	2,26	2,21

59	7003	TEAD1	TEA domain family member 1 (SV40 transcriptional enhancer factor)	2,40	1,92	2,11

60	85378	TUBGCP6	Tubulin, gamma complex associated protein 6""	2,40	2,72	2,46

61	1846	DUSP4	Dual specificity phosphatase 4	2,38	2,92	2,60

62	7422	VEGF	Vascular endothelial growth factor	2,30	2,27	2,10

63	10560	SLC19A2	Solute carrier family 19 (thiamine transporter), member 2""	2,10	2,45	2,90

64	6617	SNAPC1	Small nuclear RNA activating complex, polypeptide 1, 43 kDa""	2,05	2,65	2,38

65	6515	SLC2A3	Solute carrier family 2 (facilitated glucose transporter), member 3""	2,04	2,57	2,36

66	136	ADORA2B	Adenosine A2b receptor	1,90	1,79	1,72

FDR 10. LM: *L. monocytogenes *EGDe, SA: *S. aureus*, SP: *S. pneumoniae*.

**Table 2:** List of commonly downregulated genes for all pathogens.

				Fold Change		
**No**.	**Gene IDs**	**Symbol**	**Gene name**	**LM**	**SA**	**SP**

1	55794	DDX28	DEAD (Asp-Glu-Ala-Asp) box polypeptide 28	-11,27	-6,62	-2,56

2	9529	BAG5	BCL2-associated athanogene 5	-8,73	-6,03	-2,56

3	54554	WDR5B	WD repeat domain 5B	-8,71	-6,90	-2,98

4	80818	ZNF436	Zinc finger protein 436	-7,57	-4,44	-2,72

5	10116	FEM1B	Fem-1 homolog b (C. elegans)	-7,20	-3,67	-2,34

6	8772	FADD	Fas (TNFRSF6)-associated via death domain	-6,77	-4,31	-2,13

7	1050	CEBPA	CCAAT/enhancer binding protein (C/EBP), alpha""	-6,23	-4,57	-2,19

8	10773	ZNF482	Zinc finger protein 482	-5,98	-4,00	-2,11

9	92342	MGC9084	Chromosome 1 open reading frame 156	-5,75	-4,27	-2,74

10	51058	LOC51058	Zinc finger protein 691	-5,61	-4,22	-2,01

11	51126	NAT5	N-acetyltransferase 5 (ARD1 homolog, S. cerevisiae)""	-5,35	-5,73	-2,31

12	5718	PSMD12	Proteasome (prosome, macropain) 26S subunit, non-ATPase, 12""	-4,91	-3,51	-1,95

13	7096	TLR1	Toll-like receptor 1	-4,81	-4,18	-2,53

14	26224	FBXL3	F-box and leucine-rich repeat protein 3	-4,67	-4,09	-3,01

15	57561	ARRDC3	Arrestin domain containing 3	-4,57	-4,76	-2,36

16	148479	PHF13	PHD finger protein 13	-4,51	-3,72	-2,59

17	10978	HEAB	ATP/GTP-binding protein	-4,51	-3,24	-2,59

18	7728	ZNF175	Zinc finger protein 175	-4,50	-3,25	-2,27

19	55330	CNO	Cappuccino homolog (mouse)	-4,49	-4,60	-1,96

20	79891	FLJ23506	Zinc finger protein 671	-4,43	-3,17	-2,39

21	57547	ZNF624	Zinc finger protein 624	-3,72	-3,29	-2,26

22	7568	ZNF20	Zinc finger protein 20 (KOX 13)	-3,62	-3,80	-2,41

23	63915	TXNDC5	Thioredoxin domain containing 5	-3,57	-2,66	-2,42

24	57567	ZNF319	Zinc finger protein 319	-3,37	-4,39	-2,36

25	91574	FLJ38663	Hypothetical protein FLJ38663	-3,34	-3,20	-2,04

26	4064	LY64	CD180 antigen	-3,11	-3,23	-3,00

27	874	CBR3	Carbonyl reductase 3	-3,03	-4,09	-2,01

28	9655	SOCS5	Suppressor of cytokine signaling 5	-2,85	-2,29	-2,33

29	10668	CGRRF1	Cell growth regulator with ring finger domain 1	-2,66	-3,51	-1,92

30	8799	PEX11B	Peroxisomal biogenesis factor 11B	-2,64	-2,52	-2,44

31	132241	WDR10	Intraflagellar transport 122 homolog (Chlamydomonas)	-2,62	-2,52	-2,67

32	901	CCNG2	Cyclin G2	-2,58	-2,34	-2,53

FDR 10. LM: *L. monocytogenes *EGDe, SA: *S. aureus*, SP: *S. pneumoniae*.

Surprisingly, the major inflammatory cytokines IL1 and TNF were absent from the list of commonly expressed genes, and highest expression levels in this gene set were detected for the interleukin 23A (IL23, p19) mRNA followed by CD248 (CD164 sialomucin-like 1). In our experiment the IL23A levels were 48 -, 30- and 6-fold elevated after infection with LM, SA and SP, respectively. We observed induction of chemokines and cytokines of the CCL and CXCL families with CCL8 and CCL14 commonly induced by all thee pathogens. Genes responsible for the rearrangement of the cytoskeleton and adhesion e.g. talin2 (TLN2), claudin 1 (CLD1) and tubulin complex member protein 6 (TUBGCP6) were also upregulated (Table 1).

### Variation in the control group

The availability of non-infected controls provided us with an opportunity to assess which genes are most differently expressed among the control group before any challenge with the bacteria as well. The top most variable 100 genes were analyzed using the over-presentation analysis feature in the DAVID database (see Methods) using the entire list of accessions from the microarray chip as a background. We found that the gene categories that were highly over-represented in this set of genes (e.g. defensins B1, complement factor B, adenosine a2b receptor, inhibin) were related to the immune response. This suggests that even without bacterial challenge, gene expression of the immune response genes is highly variable and this may also reflect the different physiological state of the donors. At the other end of the spectrum, the genes with the lowest variation in gene expression were found to comprise genes responsible for cellular adhesion (integrin alpha1, tenascin, muskelin 1 etc.), cytoskeleton organization (desmin, kinesin etc.) and oxidative phosphorylation (ATP synthase subunits and cytochrome c oxidase) suggesting that maintenance of cell-cell interactions, cellular shape and energy generation are important functions that are discharged in a uniform manner across donors. The list of these most variable and the least variable genes across all donors is available as additional file (Additional file 14, Excel work sheet S2).

### Validation of microarray data by quantitative RT-PCR (qRT-PCR)

In order to verify our microarray data we performed qRT-PCR with 14 target genes. IL23A (Interleukin 23 alpha subunit, p19), JUN (Jun oncogene), NALP2 (NLR family, pyrin domain containing 2), FADD (Fas (TNFRSF6)-associated via death domain), SOCS3 (Suppressor of cytokine signaling 3), SOCS5 (Suppressor of cytokine signaling 5), TLR1 (Toll like receptor 1), SAA (Serum amyloid A2), IL21R (Interleukin 21 receptor), DEFB1 (Defensin beta 1), IL15RA (Interleukin 15 receptor, alpha), PSMB9 (Proteasome subunit beta type 9), IL10 (Interleukin 10) and INHBA (Inhibin beta A). The relative fold change of target genes was normalized by the relative expression of a pool of 4 reference genes: B2M (Beta 2 microglobulin), G6PD (Glucose 6 phosphate dehydrogenase), PGK1 (Phosphoglycerate kinase 1) and SDHA (Succinate dehydrogenase alpha subunit). Normalized fold change for a target gene versus every reference gene was calculated and a mean fold change of these four was the final value. This normalized mean fold change was plotted against the microarray expression fold change for the same target gene and the linear regression showed a correlations coefficient R^2 ^= 0.914 (Additional file 15, Figure S1).

#### IFNγ, IL12A and IL23B expression

**Figure 3:**
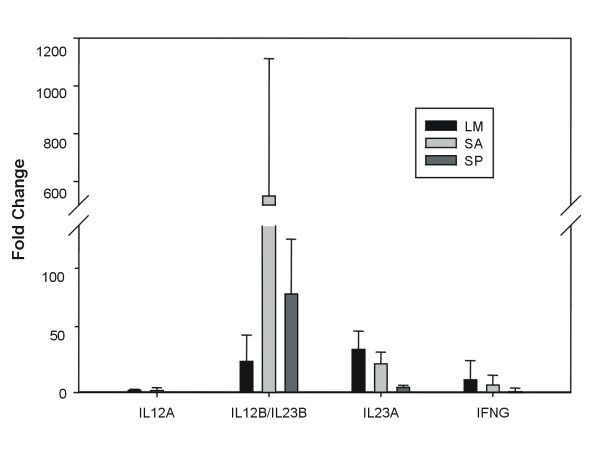
**Relative quantification of IL12A, IL12B/IL23B, IL23A and IFNγ by real time RT-PCR**. Relative expression of IL12A, IL12B/IL23B, IL23A and IFNγ (IFNG) mRNAs as determined by real time RT-PCR of infected vs. noninfected cells. Results are means plus standard deviation for all 5 donors. LM: *L. monocytogenes *EGDe, SA: *S. aureus*, SP: *S. pneumoniae.*

Since the CodeLink human UniSet I array does not contain a probe for interferon gamma (IFNγ), we additionally performed real time RT-PCR tests with IFNγ specific primers and found the mRNA to be 9.5 fold upregulated by LM, 6.2 fold induced by SA and 1.8 fold induced by SP (Figure 3; Additional file 16, Table S13). We also evaluated the relative expression of IL12A (p35) and IL23B (IL12B) mRNAs. IL12 and IL23 are heterodimeric cytokines, which share the same beta subunit, a protein of 40 KDa (IL12B/IL23B-p40). The combination of p40 with a different alpha subunit forms the physiologically active IL12 (p35p40) or IL23 (p19p40). The IL23B was not found upregulated after statistical evaluation and filtering of the primary microarray data, however IL23A (p19) mRNA was among the most strongly upregulated genes by all three pathogens and hence enhanced expression of the p40 unit was expected. The qRT-PCR data showed clearly that IL23B (IL12B) mRNA expression was increased in the monocytes of all donors. However this upregulation was highly donor-specific and varied between 2 fold and 54 fold for LM infection and reached up to more than 10^3 ^fold change for SA (Figure 3; Additional file 16, Table S13). The expression of IL12A (p35) as demonstrated by the qRT-PCR data was regulated at a much lower level with fold change values between +2 and -2 and was also donor specific.

## Discussion

Using whole-genome based microarray analysis we were able to detect the transcriptional upregulation or repression of a robust minimal set of genes in infected cells compared to untreated controls even within the short interval of one hour. Despite donor-specific gene variations and despite varying invasion strategies of the studied bacteria we identified a common program of gene expression induced by all three bacterial pathogens.

Remarkably, global comparison of the expression profiles already hinted at gross similarities by the infection among the pathogens (Figure 1, Tables 1, 2). For example, the clustering suggested that the global response of LM and SA are more similar to each other while SP infection generates a different and more subdued response pointing to similarities in the virulence of both LM and SA. One assumption may be that they generate similar responses because of their intracellular nature. However after one hour of infection we observed only a few internalized bacteria (data not shown) suggesting that secreted bacterial factors, a common feature between *L. monocytogenes *and *S. aureus *are important inducers of the response observed. LM expresses a cholesterol-dependent cytolysin (CDC) listeriolysin, that is crucial for gaining entry to the cytosol while SA encodes for several haemolysins and cytolysins e.g. the two secretory haemolysins α and β [12]. SP, on the other hand, are generally encapsulated bacteria with the capsule effectively preventing ingestion of the bacteria by the monocyte. This creates a physical barrier between the bacteria and the host cell and could underlie the observations on host gene expression made here. The similarity between pneumococcal and LM-induced gene expression could be due to the cellular response to CDC-type toxins produced by these bacteria [12]. Nevertheless, there were clear differences in the number of detectable differentially regulated genes as well, with fewer genes being differentially expressed on infection with SP. This might point to an as yet unknown mechanism for subduing the host response by SP or it might indicate the improved immune evasion ability of this particular capsular SP strain. Remarkably, hallmark inflammatory cytokines, e.g. TNF and IL1 were not part of the common response of the monocytes. However, the most prominent feature of the common genes set is the upregulation of interleukin 23A (IL23, p19) mRNA. Thus it seems that in naive human monocytes gram-positive bacteria induce the transcription of IL23 as the first major systemic proinflammatory cytokine, reminiscent of the effects of Mycobacteria and Salmonellae [13,14]. Real time RT-PCR experiments confirmed clearly the induction of IL23A (p19) mRNA as revealed by the microarray experiments. Moreover, the qRT-PCR also showed the upregulation of the IL23B (p40) gene, which however was very donor specific with a variation of 3 orders of magnitude (Figure 3; Additional file 16, Table S13). Thus infection of the monocytes with all gram-positive bacteria led to markedly increased transcription of both genes necessary to form the active IL23 cytokine. At the same time microarray as well the qRT-PCR data showed only limited upregulation or even downregulation of the IL12A (p35) mRNA transcription for the individual donors, which confirms the dominant role of IL23 versus IL12. Both IL23 and IL12 can activate the transcription activator STAT4 in Th-cells and NK cells, and stimulate the production of interferon γ (IFNγ) [15,16]. However, as the monocyte population used in this study is almost free of other leukocytes including Th-cells and NK cells, the IFNγ back loop that triggers secondary cytokine expression in the monocytes is absent, hence providing an explanation as to why other major inflammatory cytokines like IL1 and TNF were not substantially upregulated. Yet real time RT-PCR tests with IFNγ specific primers revealed upregulation of IFNγ mRNA by all three pathogens (9.5 fold upregulated by LM, 6.2 by SA and 1.8 fold by SP) suggesting an ability for self-priming of the monocytes even in the absence of additional effector cells.

The proinflammatory reaction with the hallmark upregulation of IL23 involved also the chemokines CCL8, CCL14 and osteopontin (3 to 9 fold upregulated and common for all pathogens). CCL14 weakly activates monocytes but induces the proliferation of CD34+ progenitor cells. CCL8 is chemotactic and is active on all leucocytes [17,18]. Osteopontin (SPP1, OPN) induces the migration of macrophages and dendritic cells to the site of inflammation [19]. This elevated transcription of chemokine genes is aided by the upregulation of downstream members of the inflammation signaling e.g. PDE4B, which is the predominant phosphodiesterase in macrophages and counteracts the inflammation inhibition by cyclic nucleotide signaling [20-22]. In circulating human monocytes PDE4B assists the TNFa synthesis and release in response to LPS [21,22] thus our results show that upregulation of PDE4B can also be stimulated by alternative PAMP sensors since the gram positive bacteria we have used do not produce LPS.

Recently it was discovered that bacterial LPS and non methylated CpG oligonucleotides, which signal via TLR4 and TLR9 respectively, strongly induce the expression of SOCS1 and SOCS3, and attenuate the ability of macrophages to respond to subsequent stimulation by IFNγ or IL-6 [23]. Similarly it has been described that NALP2 (PAN1) protein expression was also upregulated by LPS and interferons (IFNβ and IFNγ) and transient overexpression of NALP2 led to inhibition of NFkB signaling [24]. We also found that the upregulation of proinflammatory genes was accompanied by the upregulation of known anti-inflammatory proteins of the SOCS and NALP family (SOCS1, SOCS3, NALP2). This supports the concept of a dynamic equilibrium between inflammation induction and suppression in order to avoid excessive tissue damage. Clearly, gram-positive bacteria are also able to directly induce SOCS and NALP2 gene transcription but the actual pathway of signal transduction here must be attributed either only to TLR9 or another pathogen-recognition receptor, most likely TLR2 [25].

The microarray results also point to a novel and obviously important function of stimulated monocytes in angiogenesis and modulation of the peripheral vascular tonus. We observed the upregulation of transcription of the strong vasoactive mediators END1, VEGF and F3. Endothelin 1 (END1) is a potent vasoconstrictor and angiogenic peptide. Its expression has been attributed to damaged vascular endothelium, mast cells or macrophages in atherosclerotic lesions and thus it appears to be also a feature of stimulated monocytes in response to infection. The potential effect of endothelin induction also correlates with the upregulation of VEGF by all three pathogens. VEGF (vascular epithelium growth factor) is a major inducer of vascularization and angiogenesis [26,27]. In keeping with this observation we find that F3 (coagulation factor III thromboplastin tissue factor) is also overexpressed. Blood coagulation together with vasoconstriction ensures wound closing and prevents blood loss, but also prevents the invasion and spread of pathogens at the site of injury. Osteopontin (also upregulated) protects the endothelial cells against apoptosis and induces cell survival and proliferation. It also promotes migration of macrophages and dendritic cells to the site of inflammation and induces IL-12 secretion while down regulating the inducible nitric oxide synthase (iNOS) expression and the NO production by macrophages [19]. Our findings suggest that peripheral monocytes may have a very distinct role in processes of wound healing and the maintenance of environmental barriers when stimulated by bacterial pathogens. Interestingly some of the genes found upregulated in the monocytes were reported to have been regulated in endothelial cells upon treatment with VEGF: Egr3, Dusp4 [28] thus suggesting autocrine effects of VEGF (for LM and SA). Also the upregulation of VEGF in this study was two-fold for every single pathogen unlike the rest of upregulated cytokines and chemokines, which were usually more strongly upregulated by LM and SA. This may be interpreted as a sign for a very tight regulation of this growth factor, since another strong effect of VEGF is endothelium permeabilization, which may cause undesired exudate formation.

Another interesting characteristic of the common response was the upregulation of genes, known to counteract apoptotic signals and the absence of significant changes in the transcription of proapoptotic mediators. BIRC3 (upregulated by LM) inhibits apoptosis by binding to tumor necrosis factor receptor-associated factors TRAF1 and TRAF2 [29]. The protein encoded by TNFAIP3 is a zinc finger protein, and has been shown to inhibit TNF-mediated apoptosis [30]. Thioredoxin reductase 3 (TXNRD3) and superoxide dismutase 2 (SOD2) reduce effects of oxidative stress on mitochondria [31,32]. The upregulation of these genes indicates that the cells have to cope with higher radical production in the mitochondria caused by higher energy demands of the cell. On the other hand free radicals are produced by cytoplasmic stress and by lysosomal activity during infection. Accumulation of free radicals in the cytoplasm and in the mitochondria leads to activation of apoptotic pathways. Hence the upregulation of antiapoptotic genes and radical reducing enzymes as revealed above restores cell homeostasis and cell viability and suggests that at least at this early time point of infection the monocytes are actively suppressing an apoptotic program and are rather becoming primed for pathogen elimination and immune system activation.

The regulation of several genes was specifically influenced only by one of the pathogens. For example LM induced the transcription of FCAR (receptor for Fc fragment of IgA), a member of the immunoglobulin gene superfamily. FCAR encodes a receptor for the Fc region of IgA present on the surface of myeloid lineage cells such as neutrophils, monocytes, macrophages, and eosinophils [33]. The activated receptor triggers several immunologic defense processes, including phagocytosis, antibody-dependent cell-mediated cytotoxicity, and release of inflammatory mediators. This finding suggests the ability of the monocytes to actively interact with IgA-opsonized pathogens, which is likely to happen at entry sites of bacteria at mucosal barriers, even when the monocytes have not become tissue resident yet.

SA specifically upregulated MC1R **(**melanocortin receptor 1). The expression of MC1R on monocytes was found to be upregulated by LPS and proinflammatory cytokines [34,35]. Activation of MC1R has been shown to cause a marked reduction of activation and translocation of the nuclear transcription factor NFkB, thus suggesting that αMSH (α melanocyte stimulating hormone) exerts its anti-inflammatory effect in part through activation of MC1R [36].

Surprisingly the overall transcriptional response to infection with SP was weaker compared to LM and SA, even though this strain is a clinical isolate from an infant with severe pneumococcal pneumonia. It appearss that SP relies on its ability to avoid or delay the full innate immune response, hence the smaller number and weaker upregulation of genes involved in the initiation of inflammation (IL23, CCL8, CCL14). Also the two-fold induction of the immunosuppressive cytokine IL10 may contribute to the initial survival of the pathogen. On the other hand SP specifically induced two genes that are thought to have proinflammatory functions: CCL21 and CSF3. The protein encoded by the CCL21 gene is chemotactic *in vitro *for thymocytes and activated T cells, but not for B cells, macrophages, or neutrophils. The cytokine encoded by this gene may also play a role in mediating homing of lymphocytes to secondary lymphoid organs. CSF3 (granulocytes colony stimulation factor 3) is a cytokine that controls the production, differentiation, and function of granulocytes. We may speculate that the specific expression of the last two genes might contribute to severity of the inflammation at later stages of infection as caused by this pathogen *in vivo*.

## Conclusion

We employed DNA expression microarrays to study the early transcriptional response of naïve human peripheral monocytes infected with a set of three important gram-positive bacterial pathogens: *Staphylococcus aureus*, *Streptococcus pneumoniae *and *Listeria monocytogenes*. Upregulation of chemokine rather than interleukin genes was characteristic for the early response with the exception of the prominent expression of IL23, marking it as the lead early cytokine. An important finding was the observed activation of genes regulating angiogenesis and endothelial cell function together with genes involved in managing pathogen induced cytoplasmic stress and counteracting apoptosis. This transcription program seems to be characteristic for the first events in monocyte activation and points to induction of cytokine signalling rather than to a program change of naïve monocytes to pathogen eliminating effector cells.

## Methods

### Isolation of CD14 positive WBCs from human peripheral blood

Blood concentrates (buffy coats) were obtained routinely at the transfusion center, clinic of JLU Gießen. Approval for the use of clinical material in this study was in compliance with procedures laid down by the Helsinki Declaration and approved by the Ethics Study Board of the University Hospital of Giessen (File number 79/01). For the isolation of monocytes, only fresh (1 to 1.5 hour old) buffy coats from phenotypic healthy donors (3 males + 2 females) were used. The isolation of the mononuclear leucocytes was done by centrifugation trough a ficol cushion (Ficol-Plaque-TM, Amersham Biosciences). After the centrifugation the interphase was collected and the cells were washed twice with PBS. The cells were reconstituted in PBS and kept on ice. Anti-CD14 antibody labeled magnetic beads (Miltenyi Biotec, Bergisch Gladbach, Germany) were added to the cells in a ratio of 20 μl/10^7 ^cells (ca. 5 Abs./cell). After 15 min. incubation at 4°C unbound beads were separated by a short centrifugation step and the labeled cells were loaded and purified on a LS positive selection column using the MidiMACS magnetic separator (Miltenyi Biotec, Bergisch Gladbach, Germany) following the manufacturers instruction. The CD14+ cells were eluted in PBS and an aliquot was used for cell counting. The cells suspension was filled up with room temperetured RPMI medium (1% FCS) and portions of 5 × 10^6 ^cells/well were seated into a 6-well tissue culture dish. The cells were allowed to adhere to the plate bottom for 45 min at 37 °C in a CO_2 _tissue culture incubator.

### FACS analysis of isolated cells

Monoclonal FITC-labeled Antibodies were ordered from Miltenyi Biotec: anti CD14 clone TÜK4 and Immunotools (Friesoythe; Germany): anti CD11b-clone MEM-174. 1 μl anti CD14-FITC and 3 μl anti CD11b-FITC antibody were diluted in 50 μl of PBS, containing 0,5%BSA. 1 × 10e6 cells were added to each diluted antibody and were incubated for 30 min. at 4°C. After the incubation the cells were washed three times with 2 ml PBS/BSA by centrifugation for 5 min. at 400 g. Afterwards the cells were recovered in 0.5 ml of PBS/BSA and measured on a FACScalibure flow cytometer (BD, Heidelberg, Germany). The flow cytometer measurement revealed 12% CD14 and 28% CD11b positive cells in the mononuclear cell fraction after ficol gradient separation. The magnetic beads purified cells were enriched to 96% CD14+ and 98% CD11b+ respectively. Thus the magnetic bead separation produced a highly enriched monocyte fraction (Additional file 17, Figure S2).

### Bacterial cultures and infection assay

*L. monocytogenes *EGDe is a serotype 1/2a wild type isolate as described by Glaser P *et al. *2001 [37]. *S. aureus *Gi.11268 and *S. pneumoniae *Gi.15342 are patient isolates characterized at the Institute of Medical Microbiology, Giessen. Overnight culture of *L. monocytogenes *EGDe and *S. aureus *Gi.11268 were grown in BHI medium at 37°C by continuous shaking. The over night cultures were diluted 1:50 and bacteria were grown in BHI medium reaching an OD_600 _of 0.4 to 0.7. The number of viable bacteria was calculated using growth curves for both organisms. *S. pneumoniae *Gi.15342 was prepared by washing the bacteria with prewarmed PBS from the surface of a Columbia-agar plate with an over night Streptococcus culture. The number of viable bacteria was calculated by using a dilutions curve at OD_600. _The required bacteria were collected by centrifugation at 5000 g for 10 min. and reconstituted in RPMI medium containing 1% FCS to a final concentration of 5 × 10^7 ^bacteria/100 μl. Adherent CD14+ cells were infected by adding 100 μl of the diluted bacteria suspension yielding a moi of 10. The tissue culture plaques were swung gently to mix the infectious medium and than centrifuged for 1 min at 900 g to ensure an even contact of the bacteria with the cells. 2 to 3 control wells received 100 μl of sterile medium. The cells were incubated for 1 h in a CO_2 _tissue culture incubator followed by cell lysis and RNA isolation. No antibiotics were used by the preparation of the cells and during the infection.

### RNA isolation

For every bacterial pathogen and negative control the cells of at least two wells of a six well tissue culture plaque were lysed and total RNA was isolated. Prior to lysis culture medium was aspirated and cells lysed using RLT lysis buffer (Qiagen, Hilden, Germany). Total RNA was isolated using the RNeasy Mini Kit and the RNase free DNase I set (Qiagen) following the manufacturers protocol. The RNA was recovered in RNase free water, heat denatured for 10 min. at 65°C; quantified with the NanoDrop^® ^ND-1000 UV-Vis Spectrophotometer (NanoDrop Technologies, Rockland DE, USA) and a quality profile with the Agilent 2100 bioanalyzer (Agilent Technologies GmbH, Waldbronn, Germany) was made.

### CodeLink target labeling and array hybridization

Target preparation was done using the "CodeLink Expression Assay Reagent Kit" Manual Prep (Amersham Biosciences, Chandler AZ, USA) and the original protocol for CodeLink System manual target preparation (Amersham Biosciences, Chandler AZ, USA). Briefly: 2 μg total RNA were used in cDNA synthesis reaction with a poly-A binding primer containing the T7-polymerase promoter. Clean up of the resulting dsDNA fragments was done using the QIAquick PCR Purification Kit (Qiagen, Hilden, Germany). For target labeling the cDNA was *in vitro *transcribed by partially substituting UTP with bio-16-UTP in the reaction mixture. Labeled cRNA was purified using the RNeasy Mini Kit (Qiagen, Hilden, Germany). Portions of 20 μg cRNA were subjected to fragmentation in the presence of Mg^2+^. Subsequently 10 μg fragmented cRNA (target) was loaded onto UniSet Human I BioArray glass slides (n = 2 arrays per sample) and hybridized for 18 h in a Minitron shaker incubator (Infors AG, Bottmingen, Germany) at 37C°/300 rpm. Washing and dyeing with Cy-5 coupled streptavidin (Amersham Biosciences, Freiburg, Germany) was done according to the original protocol and the arrays were scanned using an GenePix 4000 B scanner and GenePix Pro 4.0 Software (Axon Instruments, Arlington, USA).

### Microarray data analysis

Images were analyzed using CodeLink Expression Analysis Software. Data was normalized by quantile normalization [38]. Data was log2 transformed and spots that were always flagged EMPTY were removed. Spots that were flagged empty across all technical replicates were discarded. All spots except the DISCOVERY spots were also discarded. The missing values were imputed using SeqKNN [39]. Technical replicates were averaged. Differentially expressed genes were detected using Rank Products [40], both at False Discovery Rate 5 and 10, as an unpaired analysis for each treatment being compared to the untreated control chips. The resulting gene list was subjected to DAVID and EASE [41] for annotation and overrepresentation analysis of gene categories. Due to the highly similar expression profiles of all donors to every single pathogen the microarray results presented in all tables are the mean fold change for the donor pool. The microarray data has been submitted to the ArrayExpress database and can be accessed using the accession number E-MEXP-1613.

### Real time RT-PCR

First-strand cDNA was synthesized with 500 ng of purified RNA using SuperScriptII (Invitrogen) and a mixture of T21 and random nonamer primers (Metabion) following the instructions for the reverse transcription reaction recommended for the QuantiTect SYBR Green Kit (Qiagen). Real-time quantitative PCR was performed with QuantiTect SYBR Green Kit (Qiagen) on an ABI Prism 7700 real time cycler. The relative expression of 14 target genes was normalized to that of a pool of four reference genes. PCR primers were either self-validated or commercially available QuantiTect primer assays (Qiagen). Primer sequence for the self-validated primers was as follows B2M-forward: 5'-TCTTTTTCAGTGGGGGTGA-3', B2M-reverse: 5'-TCCATCCGACATTGAAGTT-3', G6PD-forward: 5'- AGCAGTGGGGTGAAAATAC-3', G6PD-reverse: 5'-CCTGACCTACGGCAACAGA-3', TLR1-forward: 5'-TAATTTTGGATGGGCAAAGC-3', TLR1-reverse: 5'-CACCAAGTTGTCAGCGATGT-3'. For every target and reference gene a standard dilution curve with a reference RNA sample was done and the linear equation was used to transform threshold cycle values into nanograms of total RNA [42]. The relative fold change of target genes in the infected samples versus the non-treated control was normalized by the relative expression of a pool of 4 reference genes: B2M (Beta 2 microglobulin), G6PD (Glucose 6 phosphate dehydrogenase), PGK1 (Phosphoglycerate kinase 1) and SDHA (Succinate dehydrogenase alpha subunit). Normalized fold change for a target gene versus every reference gene was calculated and a mean fold change of these four was the final value.

## Authors' contributions

ST performed the experimental work and wrote the manuscript. RG participated in the statistical analysis of microarray data and in writing the manuscript. HH participated in the statistical analysis of microarray data and in writing the manuscript. TC conceived the study and helped drafting the manuscript. All authors have read and approved the final manuscript.

## Supplementary Material

Additional file 1 **Table S1**. *L. monocytogenes *- Totally upregulated genes. FDR 10. Click here for file

Additional file 2 **Table S2**. *L. monocytogenes *- Totally downregulated genes. FDR 10 Click here for file

Additional file 3 **Table S3**. *S. aureus *- Totally upregulated genes. FDR 10 Click here for file

Additional file 4 **Table S4**. *S. aureus *- Totally downregulated genes. FDR 10 Click here for file

Additional file 5 **Table S5**. *S. pneumoniae *- Totally upregulated genes. FDR 10 Click here for file

Additional file 6 **Table S6**. *S. pneumoniae *- Totally downregulated genes. FDR 10 Click here for file

Additional file 7 **Table S7**. *L. monocytogenes *- Specifically upregulated genes. FDR 10 Click here for file

Additional file 8 **Table S8**. *L. monocytogenes *- Specifically downregulated genes. FDR 10 Click here for file

Additional file 9 **Table S9**. *S. aureus *- Specifically upregulated genes. FDR 10 Click here for file

Additional file 10 **Table S10**. *S. aureus *- Specifically downregulated genes. FDR 10 Click here for file

Additional file 11 **Table S11**. *S. pneumoniae *- Specifically upregulated genes. FDR 10 Click here for file

Additional file 12 **Table S12**. *S. pneumoniae *- Specifically downregulated genes. FDR 10 Click here for file

Additional file 13 **Excel work sheet S1**. Differentially expressed genes by all three pathogens with GO grouping. Click here for file

Additional file 14 **Excel work sheet S2**. Most and least variable genes in the none challenged cells classified by Gene Ontology. Click here for file

Additional file 15 **Figure S1**. Correlation of Fold Change. Relative expression of 14 genes as determined by real time RT-PCR upon infection plotted against their corresponding microarray values. Results are averaged for all 5 donors. Click here for file

Additional file 16 **Table S13**. Relative gene expression of IL12A, IL12B/IL23B, IL23A and IFNγ, detected by real time RT-PCR Click here for file

Additional file 17 **Figure S2**. Phenotype of peripheral mononuclear cells before and after CD14+ positive selection. Anti CD11b and anti CD14 antibodies labeling after ficol gradient centrifugation and before and after CD14 positive selection. Percent of positive cells from all viable mononuclear cells. (A) CD11b + : 28% before and 98% positive cells after CD14 + selection. (B) CD14+ : 12% before and 96% positive cells after CD14 + selection Click here for file
