# Supplementary material for: Gram-positive pathogenic bacteria induce a common early response in human monocytes
Source: BMC Microbiol. 2010 Nov 2;10:275. doi: 10.1186/1471-2180-10-275 (PMC2988769; doi:10.1186/1471-2180-10-275)
Supplement: Additional file 1 — Table S1. L. monocytogenes - Totally upregulated genes. FDR 10. [file 1471-2180-10-275-S1.DOC]

**Table S1.** *L. monocytogenes* - Totally upregulated genes. FDR 10.

| **No.** | **Gene IDs** | **Gene Symbol** | **Gene Name** | **Fold Change** |
| --- | --- | --- | --- | --- |
| 1 | 51561 | IL23A | interleukin 23 alpha subunit p19 | 48,15 |
| 2 | 57124 | CD248 | CD164 sialomucin-like 1 | 20,36 |
| 3 | 2152 | F3 | coagulation factor III thromboplastin tissue factor | 17,66 |
| 4 | 3426 | IF | I factor complement | 17,07 |
| 5 | 10411 | RAPGEF3 | Rap guanine nucleotide exchange factor GEF 3 | 13,85 |
| 6 | 2272 | FHIT | fragile histidine triad gene | 12,21 |
| 7 | 1960 | EGR3 | early growth response 3 | 12,16 |
| 8 | 2669 | GEM | GTP binding protein overexpressed in skeletal muscle | 11,72 |
| 9 | 3725 | JUN | v-jun sarcoma virus 17 oncogene homolog avian | 10,80 |
| 10 | 9389 | SLC22A14 | solute carrier family 22 organic cation transporter member 14 | 10,41 |
| 11 | 6648 | SOD2 | superoxide dismutase 2 mitochondrial | 10,24 |
| 12 | 3290 | HSD11B1 | hydroxysteroid 11-beta dehydrogenase 1 | 10,04 |
| 13 | 122616 | C14orf79 | chromosome 14 open reading frame 79 | 9,27 |
| 14 | 4790 | NFKB1 | nuclear factor of kappa light polypeptide gene enhancer in B-cells 1 p105 | 8,03 |
| 15 | 131566 | DCBLD2 | discoidin CUB and LCCL domain containing 2 | 7,94 |
| 16 | 1906 | EDN1 | endothelin 1 | 7,76 |
| 17 | 9025 | RNF8 | ring finger protein C3HC4 type 8 | 7,38 |
| 18 | 51458 | RHCG | Rhesus blood group C glycoprotein | 6,90 |
| 19 | 1735 | DIO3 | deiodinase iodothyronine type III | 6,83 |
| 20 | 9021 | SOCS3 | suppressor of cytokine signaling 3 | 6,82 |
| 21 | 83740 | H2AFB3 | H2A histone family member B | 6,69 |
| 22 | 9028 | RHBDL1 | rhomboid veinlet-like 1 Drosophila | 6,18 |
| 23 | 8659 | ALDH4A1 | aldehyde dehydrogenase 4 family member A1 | 6,13 |
| 24 | 8740 | TNFSF14 | tumor necrosis factor ligand superfamily member 14 | 5,74 |
| 25 | 3908 | LAMA2 | laminin alpha 2 merosin congenital muscular dystrophy | 5,61 |
| 26 | 51025 | null | mitochondria-associated protein involved in granulocyte-macrophage colony-stimulating factor signal transduction | 5,52 |
| 27 | 10120 | ACTR1B | ARP1 actin-related protein 1 homolog B centractin beta yeast | 5,17 |
| 28 | 7128 | TNFAIP3 | tumor necrosis factor alpha-induced protein 3 | 5,13 |
| 29 | 2615 | LRRC32 | glycoprotein A repetitions predominant | 4,96 |
| 30 | 55655 | NALP2 | NACHT leucine rich repeat and PYD containing 2 | 4,72 |
| 31 | 6004 | RGS16 | regulator of G-protein signalling 16 | 4,66 |
| 32 | 6935 | TCF8 | transcription factor 8 represses interleukin 2 expression | 4,66 |
| 33 | 3280 | HES1 | hairy and enhancer of split 1 Drosophila | 4,65 |
| 34 | 983 | CDC2 | cell division cycle 2 G1 to S and G2 to M | 4,64 |
| 35 | 9076 | CLDN1 | claudin 1 | 4,60 |
| 36 | 10307 | APBB3 | amyloid beta A4 precursor protein-binding family B member 3 | 4,53 |
| 37 | 22822 | PHLDA1 | pleckstrin homology-like domain family A member 1 | 4,52 |
| 38 | 25880 | C16orf51 | DKFZP564K2062 protein | 4,36 |
| 39 | 283131 | null | null | 4,29 |
| 40 | 55567 | DNAH3 | dynein axonemal heavy polypeptide 3 | 4,17 |
| 41 | 5304 | PIP | prolactin-induced protein | 4,16 |
| 42 | 114112 | TXNRD3 | thioredoxin reductase 3 | 4,15 |
| 43 | 6355 | CCL8 | chemokine C-C motif ligand 8 | 4,06 |
| 44 | 1164 | CKS2 | CDC28 protein kinase regulatory subunit 2 | 3,95 |
| 45 | 5142 | PDE4B | phosphodiesterase 4B cAMP-specific phosphodiesterase E4 dunce homolog Drosophila | 3,93 |
| 46 | 6358 | CCL14 | chemokine C-C motif ligand 14 | 3,80 |
| 47 | 788 | SLC25A20 | solute carrier family 25 carnitine/acylcarnitine translocase member 20 | 3,72 |
| 48 | 3697 | ITIH1 | inter-alpha globulin inhibitor H1 | 3,71 |
| 49 | 57823 | SLAMF7 | SLAM family member 7 | 3,67 |
| 50 | 2322 | FLT3 | fms-related tyrosine kinase 3 | 3,66 |
| 51 | 60485 | SAV1 | salvador homolog 1 Drosophila | 3,58 |
| 52 | 6489 | ST8SIA1 | sialyltransferase 8A alpha-N-acetylneuraminate_ alpha-28-sialyltransferase GD3 synthase | 3,47 |
| 53 | 4853 | NOTCH2 | Notch homolog 2 Drosophila | 3,46 |
| 54 | 9400 | null | null | 3,38 |
| 55 | 91156 | null | eEF1A2 binding protein | 3,34 |
| 56 | 23529 | CLCF1 | cardiotrophin-like cytokine | 3,33 |
| 57 | 55647 | RAB20 | RAB20 member RAS oncogene family | 3,31 |
| 58 | 23322 | null | KIAA1005 protein | 3,31 |
| 59 | 8492 | PRSS12 | protease serine 12 neurotrypsin motopsin | 3,31 |
| 60 | 6324 | SCN1B | sodium channel voltage-gated type I beta | 3,25 |
| 61 | 6943 | TCF21 | transcription factor 21 | 3,22 |
| 62 | 54205 | CYCS | cytochrome c somatic | 3,15 |
| 63 | 54847 | SIDT1 | hypothetical protein FLJ20174 | 3,15 |
| 64 | 330 | BIRC3 | baculoviral IAP repeat-containing 3 | 3,15 |
| 65 | 3157 | HMGCS1 | 3-hydroxy-3-methylglutaryl-Coenzyme A synthase 1 soluble | 3,12 |
| 66 | 10514 | MYBBP1A | MYB binding protein P160 1a | 3,09 |
| 67 | 64108 | null | 28kD interferon responsive protein | 3,08 |
| 68 | 2569 | GABRR1 | gamma-aminobutyric acid GABA receptor rho 1 | 3,00 |
| 69 | 9590 | AKAP12 | A kinase PRKA anchor protein gravin 12 | 2,99 |
| 70 | 5894 | RAF1 | v-raf-1 murine leukemia viral oncogene homolog 1 | 2,97 |
| 71 | 7122 | CLDN5 | claudin 5 transmembrane protein deleted in velocardiofacial syndrome | 2,96 |
| 72 | 51365 | PLA1A | phospholipase A1 member A | 2,95 |
| 73 | 57176 | VARS2L | valyl-tRNA synthetase 2-like | 2,94 |
| 74 | 6696 | SPP1 | secreted phosphoprotein 1 osteopontin bone sialoprotein I early T-lymphocyte activation 1 | 2,93 |
| 75 | 348938 | null | hypothetical protein LOC348938 | 2,93 |
| 76 | 4587 | null | mucin 5 subtype B tracheobronchial | 2,92 |
| 77 | 123 | ADFP | adipose differentiation-related protein | 2,88 |
| 78 | 9451 | EIF2AK3 | eukaryotic translation initiation factor 2-alpha kinase 3 | 2,88 |
| 79 | 9819 | TSC22D2 | KIAA0669 gene product | 2,87 |
| 80 | 778 | CACNA1F | calcium channel voltage-dependent alpha 1F subunit | 2,87 |
| 81 | 54854 | null | hypothetical protein FLJ20200 | 2,84 |
| 82 | 10202 | DHRS2 | dehydrogenase/reductase SDR family member 2 | 2,84 |
| 83 | 163 | AP2B1 | adaptor-related protein complex 2 beta 1 subunit | 2,83 |
| 84 | 1939 | LGTN | ligatin | 2,80 |
| 85 | 11065 | UBE2C | ubiquitin-conjugating enzyme E2C | 2,80 |
| 86 | 57801 | HES4 | bHLH factor Hes4 | 2,78 |
| 87 | 3164 | NR4A1 | nuclear receptor subfamily 4 group A member 1 | 2,78 |
| 88 | 3202 | HOXA5 | homeo box A5 | 2,76 |
| 89 | 2304 | FOXE1 | forkhead box E1 thyroid transcription factor 2 | 2,71 |
| 90 | 2919 | CXCL1 | chemokine C-X-C motif ligand 1 melanoma growth stimulating activity alpha | 2,70 |
| 91 | 4216 | MAP3K4 | mitogen-activated protein kinase kinase kinase 4 | 2,68 |
| 92 | 80271 | ITPKC | inositol 145-trisphosphate 3-kinase C | 2,67 |
| 93 | 481 | ATP1B1 | ATPase Na+/K+ transporting beta 1 polypeptide | 2,65 |
| 94 | 1466 | CSRP2 | cysteine and glycine-rich protein 2 | 2,64 |
| 95 | 2867 | GPR43 | G protein-coupled receptor 43 | 2,64 |
| 96 | 3797 | KIF3C | kinesin family member 3C | 2,60 |
| 97 | 170680 | PSORS1C2 | psoriasis susceptibility 1 candidate 2 | 2,59 |
| 98 | 4852 | NPY | neuropeptide Y | 2,57 |
| 99 | 1847 | DUSP5 | dual specificity phosphatase 5 | 2,56 |
| 100 | 4783 | NFIL3 | nuclear factor interleukin 3 regulated | 2,55 |
| 101 | 6128 | RPL6 | ribosomal protein L6 | 2,54 |
| 102 | 55343 | SLC35C1 | solute carrier family 35 member C1 | 2,53 |
| 103 | 54840 | APTX | aprataxin | 2,53 |
| 104 | 196441 | null | hypothetical protein MGC23401 | 2,53 |
| 105 | 25822 | DNAJB5 | DnaJ Hsp40 homolog subfamily B member 5 | 2,52 |
| 106 | 733 | C8G | complement component 8 gamma polypeptide | 2,49 |
| 107 | 7056 | THBD | thrombomodulin | 2,48 |
| 108 | 11182 | SLC2A6 | solute carrier family 2 facilitated glucose transporter member 6 | 2,48 |
| 109 | 55771 | null | hypothetical protein FLJ11029 | 2,45 |
| 110 | 6580 | SLC22A1 | solute carrier family 22 organic cation transporter member 1 | 2,44 |
| 111 | 5966 | REL | v-rel reticuloendotheliosis viral oncogene homolog avian | 2,43 |
| 112 | 79073 | null | hypothetical protein MGC5508 | 2,42 |
| 113 | 83660 | TLN2 | talin 2 | 2,41 |
| 114 | 7003 | TEAD1 | TEA domain family member 1 SV40 transcriptional enhancer factor | 2,40 |
| 115 | 85378 | TUBGCP6 | tubulin gamma complex associated protein 6 | 2,40 |
| 116 | 1846 | DUSP4 | dual specificity phosphatase 4 | 2,38 |
| 117 | 6929 | TCF3 | transcription factor 3 E2A immunoglobulin enhancer binding factors E12/E47 | 2,37 |
| 118 | 5277 | PIGA | phosphatidylinositol glycan class A paroxysmal nocturnal hemoglobinuria | 2,37 |
| 119 | 8277 | TKTL1 | transketolase-like 1 | 2,35 |
| 120 | 57520 | HECW2 | NEDD4-related E3 ubiquitin ligase NEDL2 | 2,34 |
| 121 | 3656 | IRAK2 | interleukin-1 receptor-associated kinase 2 | 2,32 |
| 122 | 140453 | MUC17 | mucin 17 | 2,31 |
| 123 | 127435 | PODN | podocan | 2,31 |
| 124 | 952 | CD38 | CD38 antigen p45 | 2,31 |
| 125 | 5869 | RAB5B | RAB5B member RAS oncogene family | 2,31 |
| 126 | 55466 | DNAJA4 | DnaJ Hsp40 homolog subfamily A member 4 | 2,30 |
| 127 | 7422 | VEGF | vascular endothelial growth factor | 2,30 |
| 128 | 1827 | DSCR1 | Down syndrome critical region gene 1 | 2,28 |
| 129 | 3990 | LIPC | lipase hepatic | 2,28 |
| 130 | 5209 | PFKFB3 | 6-phosphofructo-2-kinase/fructose-26-biphosphatase 3 | 2,28 |
| 131 | 9118 | INA | internexin neuronal intermediate filament protein alpha | 2,27 |
| 132 | 1277 | COL1A1 | collagen type I alpha 1 | 2,27 |
| 133 | 1804 | DPP6 | dipeptidylpeptidase 6 | 2,26 |
| 134 | 1543 | CYP1A1 | cytochrome P450 family 1 subfamily A polypeptide 1 | 2,26 |
| 135 | 26051 | PPP1R16B | protein phosphatase 1 regulatory inhibitor subunit 16B | 2,26 |
| 136 | 320 | APBA1 | amyloid beta A4 precursor protein-binding family A member 1 X11 | 2,24 |
| 137 | 5266 | PI3 | protease inhibitor 3 skin-derived SKALP | 2,24 |
| 138 | 8651 | SOCS1 | suppressor of cytokine signaling 1 | 2,23 |
| 139 | 2618 | GART | phosphoribosylglycinamide formyltransferase phosphoribosylglycinamide synthetase phosphoribosylaminoimidazole synthetase | 2,23 |
| 140 | 27198 | GPR81 | G protein-coupled receptor 81 | 2,22 |
| 141 | 3572 | IL6ST | interleukin 6 signal transducer gp130 oncostatin M receptor | 2,20 |
| 142 | 26579 | MYEOV | myeloma overexpressed gene in a subset of t1114 positive multiple myelomas | 2,20 |
| 143 | 8756 | ADAM7 | a disintegrin and metalloproteinase domain 7 | 2,19 |
| 144 | 51186 | WBP5 | WW domain binding protein 1 | 2,18 |
| 145 | 2354 | FOSB | FBJ murine osteosarcoma viral oncogene homolog B | 2,15 |
| 146 | 399 | RHOH | ras homolog gene family member H | 2,15 |
| 147 | 3918 | LAMC2 | laminin gamma 2 | 2,14 |
| 148 | 8444 | DYRK3 | dual-specificity tyrosine-Y-phosphorylation regulated kinase 3 | 2,12 |
| 149 | 9639 | ARHGEF10 | Rho guanine nucleotide exchange factor GEF 10 | 2,11 |
| 150 | 844 | CASQ1 | calsequestrin 1 fast-twitch skeletal muscle | 2,11 |
| 151 | 9026 | null | huntingtin interacting protein-1-related | 2,11 |
| 152 | 10560 | SLC19A2 | solute carrier family 19 thiamine transporter member 2 | 2,10 |
| 153 | 2623 | GATA1 | GATA binding protein 1 globin transcription factor 1 | 2,10 |
| 154 | 11330 | CTRC | chymotrypsin C caldecrin | 2,10 |
| 155 | 55228 | null | hypothetical protein FLJ10781 | 2,07 |
| 156 | 10505 | SEMA4F | sema domain immunoglobulin domain Ig transmembrane domain TM and short cytoplasmic domain semaphorin 4F | 2,07 |
| 157 | 30819 | KCNIP2 | Kv channel interacting protein 2 | 2,06 |
| 158 | 3065 | HDAC1 | histone deacetylase 1 | 2,06 |
| 159 | 969 | CD69 | CD69 antigen p60 early T-cell activation antigen | 2,05 |
| 160 | 55340 | GIMAP5 | immune associated nucleotide 4 like 1 mouse | 2,05 |
| 161 | 26256 | CABYR | calcium-binding tyrosine-Y-phosphorylation regulated fibrousheathin 2 | 2,05 |
| 162 | 6617 | SNAPC1 | small nuclear RNA activating complex polypeptide 1 43kDa | 2,05 |
| 163 | 9256 | BZRAP1 | benzodiazapine receptor peripheral associated protein 1 | 2,04 |
| 164 | 6515 | SLC2A3 | solute carrier family 2 facilitated glucose transporter member 3 | 2,04 |
| 165 | 629 | BF | B-factor properdin | 2,04 |
| 166 | 55633 | TBC1D22B | chromosome 6 open reading frame 197 | 2,04 |
| 167 | 3158 | HMGCS2 | 3-hydroxy-3-methylglutaryl-Coenzyme A synthase 2 mitochondrial | 2,03 |
| 168 | 6288 | SAA1 | serum amyloid A1 | 2,03 |
| 169 | 27289 | RND1 | Rho family GTPase 1 | 2,02 |
| 170 | 3914 | LAMB3 | laminin beta 3 | 2,02 |
| 171 | 7466 | WFS1 | Wolfram syndrome 1 wolframin | 2,01 |
| 172 | 10265 | IRX5 | iroquois homeobox protein 5 | 2,01 |
| 173 | 23099 | ZNF297B | zinc finger protein 297B | 2,00 |
| 174 | 1875 | E2F5 | E2F transcription factor 5 p130-binding | 2,00 |
| 175 | 7062 | null | null | 2,00 |
| 176 | 23028 | AOF2 | amine oxidase flavin containing domain 2 | 1,99 |
| 177 | 55930 | MYO5C | myosin VC | 1,99 |
| 178 | 3777 | KCNK3 | potassium channel subfamily K member 3 | 1,99 |
| 179 | 786 | CACNG1 | calcium channel voltage-dependent gamma subunit 1 | 1,98 |
| 180 | 9221 | NOLC1 | nucleolar and coiled-body phosphoprotein 1 | 1,98 |
| 181 | 8424 | BBOX1 | butyrobetaine gamma 2-oxoglutarate dioxygenase gamma-butyrobetaine hydroxylase 1 | 1,97 |
| 182 | 771 | CA12 | carbonic anhydrase XII | 1,97 |
| 183 | 5989 | RFX1 | regulatory factor X 1 influences HLA class II expression | 1,96 |
| 184 | 4293 | MAP3K9 | mitogen-activated protein kinase kinase kinase 9 | 1,96 |
| 185 | 6947 | TCN1 | transcobalamin I vitamin B12 binding protein R binder family | 1,96 |
| 186 | 55311 | ZNF444 | zinc finger protein 444 | 1,96 |
| 187 | 6692 | SPINT1 | serine protease inhibitor Kunitz type 1 | 1,96 |
| 188 | 22930 | null | null | 1,95 |
| 189 | 51585 | null | pre-mRNA cleavage complex II protein Pcf11 | 1,94 |
| 190 | 4288 | MKI67 | antigen identified by monoclonal antibody Ki-67 | 1,94 |
| 191 | 4603 | MYBL1 | v-myb myeloblastosis viral oncogene homolog avian-like 1 | 1,94 |
| 192 | 22869 | ZNF510 | zinc finger protein 510 | 1,93 |
| 193 | 54535 | CCHCR1 | chromosome 6 open reading frame 18 | 1,93 |
| 194 | 50515 | CHST11 | carbohydrate chondroitin 4 sulfotransferase 11 | 1,92 |
| 195 | 10308 | ZNF267 | zinc finger protein 267 | 1,91 |
| 196 | 9262 | STK17B | serine/threonine kinase 17b apoptosis-inducing | 1,91 |
| 197 | 49854 | ZNF295 | zinc finger protein 295 | 1,90 |
| 198 | 5805 | PTS | 6-pyruvoyltetrahydropterin synthase | 1,90 |
| 199 | 1837 | DTNA | dystrobrevin alpha | 1,90 |
| 200 | 136 | ADORA2B | adenosine A2b receptor | 1,90 |
| 201 | 2204 | FCAR | Fc fragment of IgA receptor for | 1,89 |
| 202 | 11096 | ADAMTS5 | a disintegrin-like and metalloprotease reprolysin type with thrombospondin type 1 motif 5 aggrecanase-2 | 1,89 |
| 203 | 9955 | HS3ST3A1 | heparan sulfate glucosamine 3-O-sulfotransferase 3A1 | 1,78 |
| 204 | 10396 | ATP8A1 | ATPase aminophospholipid transporter APLT Class I type 8A member 1 | 1,55 |
| 205 | 171023 | null | null | 1,35 |
