# Supplementary material for: Gram-positive pathogenic bacteria induce a common early response in human monocytes
Source: BMC Microbiol. 2010 Nov 2;10:275. doi: 10.1186/1471-2180-10-275 (PMC2988769; doi:10.1186/1471-2180-10-275)
Supplement: Additional file 2 — Table S2. L. monocytogenes - Totally downregulated genes. FDR 10 [file 1471-2180-10-275-S2.DOC]

**Table S2.** *L. monocytogenes* - Totally upregulated genes. FDR 10

| **No.** | **Gene IDs** | **Gene Symbol** | **Gene Name** | **Fold Change** |
| --- | --- | --- | --- | --- |
| 1 | 55794 | DDX28 | DEAD Asp-Glu-Ala-Asp box polypeptide 28 | -11,27 |
| 2 | 9529 | BAG5 | BCL2-associated athanogene 5 | -8,73 |
| 3 | 54554 | WDR5B | WD repeat domain 5B | -8,71 |
| 4 | 80818 | ZNF436 | zinc finger protein 436 | -7,57 |
| 5 | 10116 | FEM1B | fem-1 homolog b C. elegans | -7,20 |
| 6 | 8772 | FADD | Fas TNFRSF6-associated via death domain | -6,77 |
| 7 | 1050 | CEBPA | CCAAT/enhancer binding protein C/EBP alpha | -6,23 |
| 8 | 10773 | ZNF482 | zinc finger protein 482 | -5,98 |
| 9 | 92342 | null | hypothetical protein MGC9084 | -5,75 |
| 10 | 51058 | ZNF691 | hypothetical protein LOC51058 | -5,61 |
| 11 | 6903 | TBCC | tubulin-specific chaperone c | -5,49 |
| 12 | 51126 | NAT5 | N-acetyltransferase 5 ARD1 homolog S. cerevisiae | -5,35 |
| 13 | 57665 | RDH14 | retinol dehydrogenase 14 all-trans and 9-cis | -4,95 |
| 14 | 5718 | PSMD12 | proteasome prosome macropain 26S subunit non-ATPase 12 | -4,91 |
| 15 | 7096 | TLR1 | toll-like receptor 1 | -4,81 |
| 16 | 26224 | FBXL3 | F-box and leucine-rich repeat protein 3 | -4,67 |
| 17 | 57561 | ARRDC3 | arrestin domain containing 3 | -4,57 |
| 18 | 148479 | PHF13 | PHD finger protein 13 | -4,51 |
| 19 | 10978 | null | ATP/GTP-binding protein | -4,51 |
| 20 | 7728 | ZNF175 | zinc finger protein 175 | -4,50 |
| 21 | 55330 | CNO | cappuccino | -4,49 |
| 22 | 79891 | ZNF671 | hypothetical protein FLJ23506 | -4,43 |
| 23 | 54925 | ZNF434 | zinc finger protein 434 | -4,03 |
| 24 | 29960 | FTSJ2 | FtsJ homolog 2 E. coli | -4,02 |
| 25 | 55170 | HRMT1L6 | HMT1 hnRNP methyltransferase-like 6 S. cerevisiae | -3,90 |
| 26 | 54665 | RSBN1 | hypothetical protein FLJ11220 | -3,89 |
| 27 | 57547 | ZNF624 | zinc finger protein 624 | -3,72 |
| 28 | 57215 | THAP11 | THAP domain containing 11 | -3,71 |
| 29 | 79754 | ASB13 | ankyrin repeat and SOCS box-containing 13 | -3,69 |
| 30 | 57594 | KIAA1443 | KIAA1443 | -3,64 |
| 31 | 7568 | ZNF20 | zinc finger protein 20 KOX 13 | -3,62 |
| 32 | 63915 | MUTED | muted homolog mouse | -3,57 |
| 33 | 2275 | FHL3 | four and a half LIM domains 3 | -3,55 |
| 34 | 115509 | ZNF689 | hypothetical protein BC014000 | -3,53 |
| 35 | 23361 | ZNF629 | zinc finger protein 629 | -3,48 |
| 36 | 55565 | null | hypothetical protein LOC55565 | -3,47 |
| 37 | 57567 | ZNF319 | zinc finger protein 319 | -3,37 |
| 38 | 10661 | KLF1 | Kruppel-like factor 1 erythroid | -3,35 |
| 39 | 54913 | RPP25 | ribonuclease P 25kDa subunit | -3,34 |
| 40 | 91574 | null | hypothetical protein FLJ38663 | -3,34 |
| 41 | 9841 | ZBTB24 | zinc finger and BTB domain containing 24 | -3,28 |
| 42 | 10667 | FARS2 | phenylalanine-tRNA synthetase 1 mitochondrial | -3,20 |
| 43 | 9382 | COG1 | component of oligomeric golgi complex 1 | -3,18 |
| 44 | 79177 | ZNF576 | zinc finger protein 576 | -3,17 |
| 45 | 10520 | ZNF211 | zinc finger protein 211 | -3,16 |
| 46 | 55317 | C20orf29 | chromosome 20 open reading frame 29 | -3,14 |
| 47 | 4064 | CD180 | lymphocyte antigen 64 homolog radioprotective 105kDa mouse | -3,11 |
| 48 | 81577 | null | hypothetical protein MGC11335 | -3,05 |
| 49 | 84878 | ZNF499 | zinc finger protein 499 | -3,05 |
| 50 | 874 | CBR3 | carbonyl reductase 3 | -3,03 |
| 51 | 5511 | PPP1R8 | protein phosphatase 1 regulatory inhibitor subunit 8 | -3,02 |
| 52 | 2517 | FUCA1 | fucosidase alpha-L- 1 tissue | -2,99 |
| 53 | 9823 | ARMCX2 | armadillo repeat containing X-linked 2 | -2,98 |
| 54 | 25988 | null | MBD2 methyl-CpG-binding protein-interacting zinc finger protein | -2,93 |
| 55 | 8509 | NDST2 | N-deacetylase/N-sulfotransferase heparan glucosaminyl 2 | -2,92 |
| 56 | 81572 | PDRG1 | chromosome 20 open reading frame 126 | -2,92 |
| 57 | 10363 | HMG20A | high-mobility group 20A | -2,91 |
| 58 | 1796 | DOK1 | docking protein 1 62kDa downstream of tyrosine kinase 1 | -2,88 |
| 59 | 8437 | RASAL1 | RAS protein activator like 1 GAP1 like | -2,88 |
| 60 | 10199 | MPHOSPH10 | M-phase phosphoprotein 10 U3 small nucleolar ribonucleoprotein | -2,87 |
| 61 | 550 | AUP1 | ancient ubiquitous protein 1 | -2,85 |
| 62 | 9655 | SOCS5 | suppressor of cytokine signaling 5 | -2,85 |
| 63 | 54936 | ADPRHL2 | ADP-ribosylhydrolase like 2 | -2,84 |
| 64 | 29946 | SERTAD3 | SERTA domain containing 3 | -2,78 |
| 65 | 22928 | SEPHS2 | selenophosphate synthetase 2 | -2,77 |
| 66 | 7270 | TTF1 | transcription termination factor RNA polymerase I | -2,77 |
| 67 | 7596 | ZNF45 | zinc finger protein 45 a Kruppel-associated box KRAB domain polypeptide | -2,76 |
| 68 | 9478 | CABP1 | calcium binding protein 1 calbrain | -2,75 |
| 69 | 140461 | ASB8 | ankyrin repeat and SOCS box-containing 8 | -2,71 |
| 70 | 8834 | C17orf35 | chromosome 17 open reading frame 35 | -2,69 |
| 71 | 56478 | EIF4ENIF1 | eukaryotic translation initiation factor 4E nuclear import factor 1 | -2,68 |
| 72 | 10668 | CGRRF1 | cell growth regulator with ring finger domain 1 | -2,66 |
| 73 | 2805 | GOT1 | glutamic-oxaloacetic transaminase 1 soluble aspartate aminotransferase 1 | -2,66 |
| 74 | 55299 | BXDC2 | BRIX | -2,65 |
| 75 | 8799 | PEX11B | peroxisomal biogenesis factor 11B | -2,64 |
| 76 | 51530 | ZC3HC1 | nuclear interacting partner of anaplastic lymphoma kinase ALK | -2,64 |
| 77 | 54471 | null | hypothetical protein FLJ20232 | -2,63 |
| 78 | 57645 | POGK | pogo transposable element with KRAB domain | -2,63 |
| 79 | 132241 | null | hypothetical protein LOC132241 | -2,62 |
| 80 | 10522 | DEAF1 | deformed epidermal autoregulatory factor 1 Drosophila | -2,60 |
| 81 | 10799 | RPP40 | ribonuclease P 40kDa subunit | -2,59 |
| 82 | 901 | CCNG2 | cyclin G2 | -2,58 |
| 83 | 10885 | WDR3 | WD repeat domain 3 | -2,55 |
| 84 | 50615 | IL21R | interleukin 21 receptor | -2,54 |
| 85 | 50650 | ARHGEF3 | Rho guanine nucleotide exchange factor GEF 3 | -2,52 |
| 86 | 10845 | CLPX | ClpX caseinolytic protease X homolog E. coli | -2,51 |
| 87 | 51330 | TNFRSF12A | tumor necrosis factor receptor superfamily member 12A | -2,51 |
| 88 | 25799 | ZNF324 | zinc finger protein 324 | -2,48 |
| 89 | 51067 | null | CGI-04 protein | -2,48 |
| 90 | 51250 | C6orf203 | chromosome 6 open reading frame 203 | -2,46 |
| 91 | 55039 | null | hypothetical protein FLJ20772 | -2,45 |
| 92 | 9938 | ARHGAP25 | Rho GTPase activating protein 25 | -2,43 |
| 93 | 51260 | CXorf26 | chromosome X open reading frame 26 | -2,42 |
| 94 | 150290 | DUSP18 | dual specificity phosphatase 18 | -2,41 |
| 95 | 7737 | RNF113A | zinc finger protein 183 RING finger C3HC4 type | -2,41 |
| 96 | 11244 | ZHX1 | zinc fingers and homeoboxes 1 | -2,40 |
| 97 | 81789 | TIGD6 | tigger transposable element derived 6 | -2,40 |
| 98 | 29928 | TIMM22 | translocase of inner mitochondrial membrane 22 homolog yeast | -2,40 |
| 99 | 51027 | BOLA1 | CGI-143 protein | -2,39 |
| 100 | 26269 | FBXO8 | F-box protein 8 | -2,39 |
| 101 | 23300 | null | KIAA0431 protein | -2,39 |
| 102 | 79102 | RNF26 | ring finger protein 26 | -2,39 |
| 103 | 7570 | ZNF22 | zinc finger protein 22 KOX 15 | -2,38 |
| 104 | 5279 | null | null | -2,38 |
| 105 | 79643 | CHMP6 | hypothetical protein FLJ11749 | -2,38 |
| 106 | 64146 | null | peptide deformylase-like protein | -2,38 |
| 107 | 4247 | MGAT2 | mannosyl alpha-16--glycoprotein beta-12-N-acetylglucosaminyltransferase | -2,36 |
| 108 | 23580 | CDC42EP4 | CDC42 effector protein Rho GTPase binding 4 | -2,35 |
| 109 | 81488 | GRINL1A | glutamate receptor ionotropic N-methyl D-aspartate-like 1A | -2,35 |
| 110 | 56342 | PPAN | peter pan homolog Drosophila | -2,34 |
| 111 | 11334 | TUSC2 | tumor suppressor candidate 2 | -2,34 |
| 112 | 9595 | PSCDBP | pleckstrin homology Sec7 and coiled-coil domains binding protein | -2,34 |
| 113 | 11218 | DDX20 | DEAD Asp-Glu-Ala-Asp box polypeptide 20 | -2,34 |
| 114 | 29914 | null | transitional epithelia response protein | -2,33 |
| 115 | 7741 | ZNF187 | zinc finger protein 187 | -2,33 |
| 116 | 55180 | LINS1 | WINS1 protein with Drosophila Lines Lin homologous domain | -2,32 |
| 117 | 7533 | YWHAH | tyrosine 3-monooxygenase/tryptophan 5-monooxygenase activation protein eta polypeptide | -2,31 |
| 118 | 54826 | null | hypothetical protein FLJ20125 | -2,30 |
| 119 | 55153 | SDAD1 | SDA1 domain containing 1 | -2,30 |
| 120 | 5624 | PROC | protein C inactivator of coagulation factors Va and VIIIa | -2,30 |
| 121 | 5031 | P2RY6 | pyrimidinergic receptor P2Y G-protein coupled 6 | -2,30 |
| 122 | 8846 | ALKBH | alkB alkylation repair homolog E. coli | -2,30 |
| 123 | 79724 | null | hypothetical protein FLJ23436 | -2,30 |
| 124 | 23463 | ICMT | isoprenylcysteine carboxyl methyltransferase | -2,29 |
| 125 | 7268 | TTC4 | tetratricopeptide repeat domain 4 | -2,29 |
| 126 | 669 | BPGM | 23-bisphosphoglycerate mutase | -2,28 |
| 127 | 7584 | ZNF35 | zinc finger protein 35 clone HF.10 | -2,27 |
| 128 | 1052 | CEBPD | CCAAT/enhancer binding protein C/EBP delta | -2,27 |
| 129 | 55145 | THAP1 | THAP domain containing apoptosis associated protein 1 | -2,26 |
| 130 | 7764 | ZNF217 | zinc finger protein 217 | -2,25 |
| 131 | 26164 | GTPBP5 | GTP binding protein 5 putative | -2,25 |
| 132 | 9049 | AIP | aryl hydrocarbon receptor interacting protein | -2,23 |
| 133 | 7132 | TNFRSF1A | tumor necrosis factor receptor superfamily member 1A | -2,23 |
| 134 | 51147 | ING4 | inhibitor of growth family member 4 | -2,23 |
| 135 | 5977 | DPF2 | D4 zinc and double PHD fingers family 2 | -2,21 |
| 136 | 26127 | FGFR1OP2 | FGFR1 oncogene partner 2 | -2,21 |
| 137 | 26586 | CKAP2 | cytoskeleton associated protein 2 | -2,20 |
| 138 | 51193 | ZNF639 | zinc finger protein 639 | -2,20 |
| 139 | 6513 | SLC2A1 | solute carrier family 2 facilitated glucose transporter member 1 | -2,20 |
| 140 | 55339 | WDR33 | WD repeat domain 33 | -2,20 |
| 141 | 11266 | DUSP12 | dual specificity phosphatase 12 | -2,20 |
| 142 | 9724 | UTP14C | UTP14 U3 small nucleolar ribonucleoprotein homolog C yeast | -2,19 |
| 143 | 23067 | null | KIAA1076 protein | -2,19 |
| 144 | 53838 | C11orf24 | chromosome 11 open reading frame 24 | -2,19 |
| 145 | 23483 | TGDS | TDP-glucose 46-dehydratase | -2,19 |
| 146 | 5194 | PEX13 | peroxisome biogenesis factor 13 | -2,18 |
| 147 | 8455 | null | null | -2,18 |
| 148 | 29090 | C18orf55 | HSPC154 protein | -2,18 |
| 149 | 7398 | USP1 | ubiquitin specific protease 1 | -2,18 |
| 150 | 59348 | ZNF350 | zinc finger protein 350 | -2,17 |
| 151 | 54861 | SNRK | SNF-1 related kinase | -2,17 |
| 152 | 1974 | EIF4A2 | eukaryotic translation initiation factor 4A isoform 2 | -2,17 |
| 153 | 26499 | PLEK2 | pleckstrin 2 | -2,17 |
| 154 | 10128 | LRPPRC | leucine-rich PPR-motif containing | -2,16 |
| 155 | 51523 | CXXC5 | CXXC finger 5 | -2,16 |
| 156 | 6195 | RPS6KA1 | ribosomal protein S6 kinase 90kDa polypeptide 1 | -2,16 |
| 157 | 9534 | ZNF254 | zinc finger protein 254 | -2,16 |
| 158 | 81873 | ARPC5L | actin related protein 2/3 complex subunit 5-like | -2,15 |
| 159 | 1605 | DAG1 | dystroglycan 1 dystrophin-associated glycoprotein 1 | -2,15 |
| 160 | 7343 | UBTF | upstream binding transcription factor RNA polymerase I | -2,15 |
| 161 | 51601 | LIPT1 | lipoyltransferase 1 | -2,15 |
| 162 | 51499 | null | hypothetical protein HSPC132 | -2,14 |
| 163 | 23212 | RRS1 | RRS1 ribosome biogenesis regulator homolog S. cerevisiae | -2,14 |
| 164 | 3396 | ICT1 | immature colon carcinoma transcript 1 | -2,14 |
| 165 | 7884 | SLBP | stem-loop histone binding protein | -2,12 |
| 166 | 5929 | RBBP5 | retinoblastoma binding protein 5 | -2,12 |
| 167 | 60496 | AASDHPPT | aminoadipate-semialdehyde dehydrogenase-phosphopantetheinyl transferase | -2,12 |
| 168 | 26168 | SENP3 | SUMO1/sentrin/SMT3 specific protease 3 | -2,12 |
| 169 | 4690 | NCK1 | NCK adaptor protein 1 | -2,12 |
| 170 | 9868 | TOMM70A | translocase of outer mitochondrial membrane 70 homolog A yeast | -2,11 |
| 171 | 580 | BARD1 | BRCA1 associated RING domain 1 | -2,11 |
| 172 | 54989 | null | hypothetical protein FLJ20582 | -2,10 |
| 173 | 29915 | HCFC2 | host cell factor C2 | -2,10 |
| 174 | 5998 | null | null | -2,09 |
| 175 | 10458 | BAIAP2 | BAI1-associated protein 2 | -2,09 |
| 176 | 55030 | FBXO34 | F-box protein 34 | -2,09 |
| 177 | 54957 | TXNL4B | Dim1-like protein | -2,07 |
| 178 | 7551 | ZNF3 | zinc finger protein 3 A8-51 | -2,07 |
| 179 | 54851 | null | fetal globin-inducing factor | -2,06 |
| 180 | 11108 | PRDM4 | PR domain containing 4 | -2,06 |
| 181 | 55602 | null | collaborates/cooperates with ARF alternate reading frame protein | -2,06 |
| 182 | 400657 | null | hypothetical LOC400657 | -2,05 |
| 183 | 11277 | TREX1 | three prime repair exonuclease 1 | -2,05 |
| 184 | 6049 | RNF6 | ring finger protein C3H2C3 type 6 | -2,05 |
| 185 | 1789 | DNMT3B | DNA cytosine-5--methyltransferase 3 beta | -2,05 |
| 186 | 53918 | PELO | pelota homolog Drosophila | -2,03 |
| 187 | 23438 | HARSL | histidyl-tRNA synthetase-like | -2,03 |
| 188 | 5134 | PDCD2 | programmed cell death 2 | -2,03 |
| 189 | 51244 | C3orf19 | hypothetical protein LOC51244 | -2,03 |
| 190 | 8131 | C16orf35 | chromosome 16 open reading frame 35 | -2,03 |
| 191 | 10884 | MRPS30 | mitochondrial ribosomal protein S30 | -2,02 |
| 192 | 10693 | CCT6B | chaperonin containing TCP1 subunit 6B zeta 2 | -2,02 |
| 193 | 55182 | null | hypothetical protein FLJ10597 | -2,02 |
| 194 | 10044 | SH2D3C | SH2 domain containing 3C | -2,02 |
| 195 | 10633 | null | RAS-related on chromosome 22 | -2,02 |
| 196 | 10322 | SMYD5 | SMYD family member 5 | -2,01 |
| 197 | 54849 | null | null | -2,01 |
| 198 | 10084 | PQBP1 | polyglutamine binding protein 1 | -2,01 |
| 199 | 7727 | ZNF174 | zinc finger protein 174 | -2,01 |
| 200 | 9668 | ZNF432 | zinc finger protein 432 | -2,01 |
| 201 | 64785 | null | hypothetical protein FLJ13912 | -2,00 |
| 202 | 5695 | PSMB7 | proteasome prosome macropain subunit beta type 7 | -2,00 |
| 203 | 54785 | null | hypothetical protein FLJ20014 | -2,00 |
| 204 | 9889 | ZBED4 | zinc finger BED domain containing 4 | -2,00 |
| 205 | 9183 | ZW10 | ZW10 homolog centromere/kinetochore protein Drosophila | -1,99 |
| 206 | 80019 | UBTD1 | ubiquitin domain containing 1 | -1,99 |
| 207 | 5111 | PCNA | proliferating cell nuclear antigen | -1,99 |
| 208 | 57542 | null | null | -1,99 |
| 209 | 10795 | null | null | -1,98 |
| 210 | 10023 | FRAT1 | frequently rearranged in advanced T-cell lymphomas | -1,97 |
| 211 | 8364 | HIST1H4C | histone 1 H4c | -1,97 |
| 212 | 7358 | UGDH | UDP-glucose dehydrogenase | -1,97 |
| 213 | 55352 | null | hypothetical protein clone 2746033 | -1,97 |
| 214 | 4066 | LYL1 | lymphoblastic leukemia derived sequence 1 | -1,97 |
| 215 | 10626 | TRIM16 | tripartite motif-containing 16 | -1,97 |
| 216 | 2214 | FCGR3A | Fc fragment of IgG low affinity IIIa receptor for CD16 | -1,96 |
| 217 | 56985 | null | x 006 protein | -1,96 |
| 218 | 333926 | PPM1J | hypothetical protein MGC19531 | -1,96 |
| 219 | 11124 | FAF1 | Fas TNFRSF6 associated factor 1 | -1,95 |
| 220 | 9046 | DOK2 | docking protein 2 56kDa | -1,95 |
| 221 | 55905 | ZNF313 | zinc finger protein 313 | -1,94 |
| 222 | 10215 | OLIG2 | oligodendrocyte lineage transcription factor 2 | -1,91 |
| 223 | 55113 | null | hypothetical protein FLJ10307 | -1,90 |
| 224 | 57569 | ARHGAP20 | Rho GTPase activating protein 20 | -1,88 |
| 225 | 10206 | null | null | -1,81 |
| 226 | 6194 | RPS6 | ribosomal protein S6 | -1,76 |
| 227 | 55658 | RNF126 | ring finger protein 126 | -1,75 |
| 228 | 57479 | null | null | -1,73 |
| 229 | 4702 | NDUFA8 | NADH dehydrogenase ubiquinone 1 alpha subcomplex 8 19kDa | -1,57 |
| 230 | 713 | C1QB | complement component 1 q subcomponent beta polypeptide | -1,52 |
| 231 | 7436 | null | null | -1,24 |
| 232 | 9659 | PDE4DIP | phosphodiesterase 4D interacting protein myomegalin | -1,10 |
| 233 | 8284 | SMCY | Jumonji AT rich interactive domain 1D RBP2-like | -1,08 |
