# Supplementary material for: Gram-positive pathogenic bacteria induce a common early response in human monocytes
Source: BMC Microbiol. 2010 Nov 2;10:275. doi: 10.1186/1471-2180-10-275 (PMC2988769; doi:10.1186/1471-2180-10-275)
Supplement: Additional file 4 — Table S4. S. aureus - Totally downregulated genes. FDR 10 [file 1471-2180-10-275-S4.DOC]

**Table S4.** *S. aureus –* Totally downregulated genes. FDR 10.

| **No.** | **Gene IDs** | **Gene Symbol** | **Gene Name** | **Fold Change** |
| --- | --- | --- | --- | --- |
| 1 | 54554 | WDR5B | WD repeat domain 5B | -6,90 |
| 2 | 55794 | DDX28 | DEAD Asp-Glu-Ala-Asp box polypeptide 28 | -6,62 |
| 3 | 9529 | BAG5 | BCL2-associated athanogene 5 | -6,03 |
| 4 | 51126 | NAT5 | N-acetyltransferase 5 ARD1 homolog S. cerevisiae | -5,73 |
| 5 | 57561 | ARRDC3 | arrestin domain containing 3 | -4,76 |
| 6 | 55330 | CNO | cappuccino | -4,60 |
| 7 | 1050 | CEBPA | CCAAT/enhancer binding protein C/EBP alpha | -4,57 |
| 8 | 80818 | ZNF436 | zinc finger protein 436 | -4,44 |
| 9 | 57567 | ZNF319 | zinc finger protein 319 | -4,39 |
| 10 | 6903 | TBCC | tubulin-specific chaperone c | -4,32 |
| 11 | 8772 | FADD | Fas TNFRSF6-associated via death domain | -4,31 |
| 12 | 92342 | null | hypothetical protein MGC9084 | -4,27 |
| 13 | 51058 | ZNF691 | hypothetical protein LOC51058 | -4,22 |
| 14 | 7096 | TLR1 | toll-like receptor 1 | -4,18 |
| 15 | 26224 | FBXL3 | F-box and leucine-rich repeat protein 3 | -4,09 |
| 16 | 874 | CBR3 | carbonyl reductase 3 | -4,09 |
| 17 | 10773 | ZNF482 | zinc finger protein 482 | -4,00 |
| 18 | 7568 | ZNF20 | zinc finger protein 20 KOX 13 | -3,80 |
| 19 | 148479 | PHF13 | PHD finger protein 13 | -3,72 |
| 20 | 10116 | FEM1B | fem-1 homolog b C. elegans | -3,67 |
| 21 | 25988 | null | MBD2 methyl-CpG-binding protein-interacting zinc finger protein | -3,64 |
| 22 | 5718 | PSMD12 | proteasome prosome macropain 26S subunit non-ATPase 12 | -3,51 |
| 23 | 10668 | CGRRF1 | cell growth regulator with ring finger domain 1 | -3,51 |
| 24 | 115509 | ZNF689 | hypothetical protein BC014000 | -3,40 |
| 25 | 79177 | ZNF576 | zinc finger protein 576 | -3,37 |
| 26 | 57665 | RDH14 | retinol dehydrogenase 14 all-trans and 9-cis | -3,36 |
| 27 | 84878 | ZNF499 | zinc finger protein 499 | -3,32 |
| 28 | 57547 | ZNF624 | zinc finger protein 624 | -3,29 |
| 29 | 57594 | KIAA1443 | KIAA1443 | -3,26 |
| 30 | 7728 | ZNF175 | zinc finger protein 175 | -3,25 |
| 31 | 81577 | null | hypothetical protein MGC11335 | -3,24 |
| 32 | 10978 | null | ATP/GTP-binding protein | -3,24 |
| 33 | 4064 | CD180 | lymphocyte antigen 64 homolog radioprotective 105kDa mouse | -3,23 |
| 34 | 91574 | null | hypothetical protein FLJ38663 | -3,20 |
| 35 | 79891 | ZNF671 | hypothetical protein FLJ23506 | -3,17 |
| 36 | 9724 | UTP14C | UTP14 U3 small nucleolar ribonucleoprotein homolog C yeast | -3,16 |
| 37 | 2517 | FUCA1 | fucosidase alpha-L- 1 tissue | -3,07 |
| 38 | 51601 | LIPT1 | lipoyltransferase 1 | -3,03 |
| 39 | 10661 | KLF1 | Kruppel-like factor 1 erythroid | -2,99 |
| 40 | 81572 | PDRG1 | chromosome 20 open reading frame 126 | -2,99 |
| 41 | 29915 | HCFC2 | host cell factor C2 | -2,95 |
| 42 | 29946 | SERTAD3 | SERTA domain containing 3 | -2,95 |
| 43 | 9841 | ZBTB24 | zinc finger and BTB domain containing 24 | -2,87 |
| 44 | 51275 | null | apoptosis-related protein PNAS-1 | -2,87 |
| 45 | 55170 | HRMT1L6 | HMT1 hnRNP methyltransferase-like 6 S. cerevisiae | -2,82 |
| 46 | 5698 | PSMB9 | proteasome prosome macropain subunit beta type 9 large multifunctional protease 2 | -2,81 |
| 47 | 29928 | TIMM22 | translocase of inner mitochondrial membrane 22 homolog yeast | -2,80 |
| 48 | 57215 | THAP11 | THAP domain containing 11 | -2,77 |
| 49 | 54665 | RSBN1 | hypothetical protein FLJ11220 | -2,76 |
| 50 | 2275 | FHL3 | four and a half LIM domains 3 | -2,75 |
| 51 | 51027 | BOLA1 | CGI-143 protein | -2,74 |
| 52 | 55039 | null | hypothetical protein FLJ20772 | -2,73 |
| 53 | 8834 | C17orf35 | chromosome 17 open reading frame 35 | -2,72 |
| 54 | 79754 | ASB13 | ankyrin repeat and SOCS box-containing 13 | -2,71 |
| 55 | 25949 | CBPIN | GCIP-interacting protein p29 | -2,70 |
| 56 | 54471 | null | hypothetical protein FLJ20232 | -2,69 |
| 57 | 54957 | TXNL4B | Dim1-like protein | -2,68 |
| 58 | 10633 | null | RAS-related on chromosome 22 | -2,68 |
| 59 | 63915 | MUTED | muted homolog mouse | -2,66 |
| 60 | 51147 | ING4 | inhibitor of growth family member 4 | -2,66 |
| 61 | 51499 | null | hypothetical protein HSPC132 | -2,65 |
| 62 | 29960 | FTSJ2 | FtsJ homolog 2 E. coli | -2,64 |
| 63 | 9478 | CABP1 | calcium binding protein 1 calbrain | -2,62 |
| 64 | 51250 | C6orf203 | chromosome 6 open reading frame 203 | -2,60 |
| 65 | 5194 | PEX13 | peroxisome biogenesis factor 13 | -2,59 |
| 66 | 51260 | CXorf26 | chromosome X open reading frame 26 | -2,58 |
| 67 | 81789 | TIGD6 | tigger transposable element derived 6 | -2,56 |
| 68 | 8455 | null | null | -2,56 |
| 69 | 11018 | TMED1 | interleukin 1 receptor-like 1 ligand | -2,56 |
| 70 | 5695 | PSMB7 | proteasome prosome macropain subunit beta type 7 | -2,56 |
| 71 | 8799 | PEX11B | peroxisomal biogenesis factor 11B | -2,52 |
| 72 | 23361 | ZNF629 | zinc finger protein 629 | -2,52 |
| 73 | 132241 | null | hypothetical protein LOC132241 | -2,52 |
| 74 | 51067 | null | CGI-04 protein | -2,52 |
| 75 | 130916 | null | hypothetical protein MGC61716 | -2,48 |
| 76 | 55180 | LINS1 | WINS1 protein with Drosophila Lines Lin homologous domain | -2,46 |
| 77 | 1605 | DAG1 | dystroglycan 1 dystrophin-associated glycoprotein 1 | -2,46 |
| 78 | 29100 | null | HSPC171 protein | -2,44 |
| 79 | 7551 | ZNF3 | zinc finger protein 3 A8-51 | -2,43 |
| 80 | 2992 | GYG | glycogenin | -2,42 |
| 81 | 81873 | ARPC5L | actin related protein 2/3 complex subunit 5-like | -2,42 |
| 82 | 10667 | FARS2 | phenylalanine-tRNA synthetase 1 mitochondrial | -2,42 |
| 83 | 55145 | THAP1 | THAP domain containing apoptosis associated protein 1 | -2,41 |
| 84 | 55272 | C15orf12 | chromosome 15 open reading frame 12 | -2,41 |
| 85 | 55317 | C20orf29 | chromosome 20 open reading frame 29 | -2,41 |
| 86 | 6341 | SCO1 | SCO cytochrome oxidase deficient homolog 1 yeast | -2,41 |
| 87 | 55299 | BXDC2 | BRIX | -2,38 |
| 88 | 79724 | null | hypothetical protein FLJ23436 | -2,35 |
| 89 | 51330 | TNFRSF12A | tumor necrosis factor receptor superfamily member 12A | -2,35 |
| 90 | 901 | CCNG2 | cyclin G2 | -2,34 |
| 91 | 55352 | null | hypothetical protein clone 2746033 | -2,33 |
| 92 | 26586 | CKAP2 | cytoskeleton associated protein 2 | -2,33 |
| 93 | 54913 | RPP25 | ribonuclease P 25kDa subunit | -2,32 |
| 94 | 54849 | null | null | -2,32 |
| 95 | 3396 | ICT1 | immature colon carcinoma transcript 1 | -2,32 |
| 96 | 9668 | ZNF432 | zinc finger protein 432 | -2,32 |
| 97 | 7533 | YWHAH | tyrosine 3-monooxygenase/tryptophan 5-monooxygenase activation protein eta polypeptide | -2,30 |
| 98 | 7559 | null | null | -2,30 |
| 99 | 9412 | SURB7 | SRB7 suppressor of RNA polymerase B homolog yeast | -2,29 |
| 100 | 9655 | SOCS5 | suppressor of cytokine signaling 5 | -2,29 |
| 101 | 10799 | RPP40 | ribonuclease P 40kDa subunit | -2,29 |
| 102 | 7584 | ZNF35 | zinc finger protein 35 clone HF.10 | -2,28 |
| 103 | 55113 | null | hypothetical protein FLJ10307 | -2,27 |
| 104 | 4702 | NDUFA8 | NADH dehydrogenase ubiquinone 1 alpha subcomplex 8 19kDa | -2,26 |
| 105 | 59348 | ZNF350 | zinc finger protein 350 | -2,25 |
| 106 | 1789 | DNMT3B | DNA cytosine-5--methyltransferase 3 beta | -2,24 |
| 107 | 23438 | HARSL | histidyl-tRNA synthetase-like | -2,23 |
| 108 | 90407 | TMEM41A | similar to RIKEN cDNA 5730578N08 gene | -2,23 |
| 109 | 11218 | DDX20 | DEAD Asp-Glu-Ala-Asp box polypeptide 20 | -2,21 |
| 110 | 5111 | PCNA | proliferating cell nuclear antigen | -2,20 |
| 111 | 7905 | C5orf18 | chromosome 5 open reading frame 18 | -2,20 |
| 112 | 1796 | DOK1 | docking protein 1 62kDa downstream of tyrosine kinase 1 | -2,19 |
| 113 | 25940 | null | DKFZP564F0522 protein | -2,19 |
| 114 | 7741 | ZNF187 | zinc finger protein 187 | -2,19 |
| 115 | 4066 | LYL1 | lymphoblastic leukemia derived sequence 1 | -2,19 |
| 116 | 11344 | PTK9L | PTK9L protein tyrosine kinase 9-like A6-related protein | -2,19 |
| 117 | 9868 | TOMM70A | translocase of outer mitochondrial membrane 70 homolog A yeast | -2,18 |
| 118 | 54785 | null | hypothetical protein FLJ20014 | -2,18 |
| 119 | 9587 | MAD2L1BP | MAD2L1 binding protein | -2,17 |
| 120 | 54989 | null | hypothetical protein FLJ20582 | -2,17 |
| 121 | 9049 | AIP | aryl hydrocarbon receptor interacting protein | -2,16 |
| 122 | 9382 | COG1 | component of oligomeric golgi complex 1 | -2,16 |
| 123 | 8629 | JRK | jerky homolog mouse | -2,16 |
| 124 | 57645 | POGK | pogo transposable element with KRAB domain | -2,16 |
| 125 | 9595 | PSCDBP | pleckstrin homology Sec7 and coiled-coil domains binding protein | -2,16 |
| 126 | 29090 | C18orf55 | HSPC154 protein | -2,15 |
| 127 | 23463 | ICMT | isoprenylcysteine carboxyl methyltransferase | -2,15 |
| 128 | 26098 | C10orf137 | chromosome 10 open reading frame 137 | -2,15 |
| 129 | 64146 | null | peptide deformylase-like protein | -2,15 |
| 130 | 54925 | ZNF434 | zinc finger protein 434 | -2,15 |
| 131 | 10199 | MPHOSPH10 | M-phase phosphoprotein 10 U3 small nucleolar ribonucleoprotein | -2,14 |
| 132 | 23483 | TGDS | TDP-glucose 46-dehydratase | -2,13 |
| 133 | 25799 | ZNF324 | zinc finger protein 324 | -2,13 |
| 134 | 140461 | ASB8 | ankyrin repeat and SOCS box-containing 8 | -2,12 |
| 135 | 81689 | HBLD2 | HESB like domain containing 2 | -2,12 |
| 136 | 550 | AUP1 | ancient ubiquitous protein 1 | -2,11 |
| 137 | 55565 | null | hypothetical protein LOC55565 | -2,10 |
| 138 | 84337 | ELOF1 | hypothetical protein MGC4549 | -2,10 |
| 139 | 23067 | null | KIAA1076 protein | -2,09 |
| 140 | 56478 | EIF4ENIF1 | eukaryotic translation initiation factor 4E nuclear import factor 1 | -2,08 |
| 141 | 51023 | MRPS18C | mitochondrial ribosomal protein S18C | -2,08 |
| 142 | 1477 | CSTF1 | cleavage stimulation factor 3 pre-RNA subunit 1 50kDa | -2,08 |
| 143 | 26164 | GTPBP5 | GTP binding protein 5 putative | -2,07 |
| 144 | 6941 | TCF19 | transcription factor 19 SC1 | -2,06 |
| 145 | 54936 | ADPRHL2 | ADP-ribosylhydrolase like 2 | -2,06 |
| 146 | 80778 | ZNF34 | zinc finger protein 34 KOX 32 | -2,06 |
| 147 | 10363 | HMG20A | high-mobility group 20A | -2,05 |
| 148 | 57150 | C6orf162 | chromosome 6 open reading frame 162 | -2,04 |
| 149 | 8976 | WASL | Wiskott-Aldrich syndrome-like | -2,03 |
| 150 | 6118 | RPA2 | replication protein A2 32kDa | -2,02 |
| 151 | 26157 | GIMAP2 | immunity associated protein 2 | -2,01 |
| 152 | 27440 | CECR5 | cat eye syndrome chromosome region candidate 5 | -2,01 |
| 153 | 5998 | null | null | -2,01 |
| 154 | 580 | BARD1 | BRCA1 associated RING domain 1 | -2,00 |
| 155 | 7727 | ZNF174 | zinc finger protein 174 | -2,00 |
| 156 | 10102 | TSFM | Ts translation elongation factor mitochondrial | -1,99 |
| 157 | 51106 | TFB1M | transcription factor B1 mitochondrial | -1,98 |
| 158 | 9823 | ARMCX2 | armadillo repeat containing X-linked 2 | -1,97 |
| 159 | 10206 | null | null | -1,95 |
| 160 | 50650 | ARHGEF3 | Rho guanine nucleotide exchange factor GEF 3 | -1,95 |
| 161 | 57864 | TSCOT | thymic stromal co-transporter | -1,88 |
| 162 | 5624 | PROC | protein C inactivator of coagulation factors Va and VIIIa | -1,88 |
| 163 | 974 | CD79B | CD79B antigen immunoglobulin-associated beta | -1,86 |
| 164 | 26499 | PLEK2 | pleckstrin 2 | -1,86 |
| 165 | 79643 | CHMP6 | hypothetical protein FLJ11749 | -1,85 |
| 166 | 51193 | ZNF639 | zinc finger protein 639 | -1,85 |
| 167 | 6194 | RPS6 | ribosomal protein S6 | -1,84 |
| 168 | 55303 | GIMAP4 | immunity associated protein 4 | -1,80 |
| 169 | 114882 | OSBPL8 | oxysterol binding protein-like 8 | -1,69 |
| 170 | 713 | C1QB | complement component 1 q subcomponent beta polypeptide | -1,54 |
| 171 | 9167 | COX7A2L | cytochrome c oxidase subunit VIIa polypeptide 2 like | -1,43 |
