# Supplementary material for: Gram-positive pathogenic bacteria induce a common early response in human monocytes
Source: BMC Microbiol. 2010 Nov 2;10:275. doi: 10.1186/1471-2180-10-275 (PMC2988769; doi:10.1186/1471-2180-10-275)
Supplement: Additional file 5 — Table S5. S. pneumoniae - Totally upregulated genes. FDR 10 [file 1471-2180-10-275-S5.DOC]

**Table S5.** *S. pneumoniae* - Totally upregulated genes. FDR 10.

| **No.** | **Gene IDs** | **Gene Symbol** | **Gene Name** | **Fold Change** |
| --- | --- | --- | --- | --- |
| 1 | 89941 | RHOT2 | ras homolog gene family member T2 | 22,18 |
| 2 | 3624 | INHBA | inhibin beta A activin A activin AB alpha polypeptide | 8,55 |
| 3 | 8013 | NR4A3 | nuclear receptor subfamily 4 group A member 3 | 8,40 |
| 4 | 57124 | CD248 | CD164 sialomucin-like 1 | 8,23 |
| 5 | 6935 | TCF8 | transcription factor 8 represses interleukin 2 expression | 7,11 |
| 6 | 3426 | IF | I factor complement | 6,88 |
| 7 | 51561 | IL23A | interleukin 23 alpha subunit p19 | 6,70 |
| 8 | 3725 | JUN | v-jun sarcoma virus 17 oncogene homolog avian | 6,35 |
| 9 | 3290 | HSD11B1 | hydroxysteroid 11-beta dehydrogenase 1 | 5,73 |
| 10 | 10411 | RAPGEF3 | Rap guanine nucleotide exchange factor GEF 3 | 5,60 |
| 11 | 2272 | FHIT | fragile histidine triad gene | 5,48 |
| 12 | 9389 | SLC22A14 | solute carrier family 22 organic cation transporter member 14 | 5,40 |
| 13 | 131566 | DCBLD2 | discoidin CUB and LCCL domain containing 2 | 4,59 |
| 14 | 122616 | C14orf79 | chromosome 14 open reading frame 79 | 4,45 |
| 15 | 6648 | SOD2 | superoxide dismutase 2 mitochondrial | 4,23 |
| 16 | 55647 | RAB20 | RAB20 member RAS oncogene family | 3,95 |
| 17 | 2669 | GEM | GTP binding protein overexpressed in skeletal muscle | 3,90 |
| 18 | 6358 | CCL14 | chemokine C-C motif ligand 14 | 3,76 |
| 19 | 1735 | DIO3 | deiodinase iodothyronine type III | 3,70 |
| 20 | 1466 | CSRP2 | cysteine and glycine-rich protein 2 | 3,66 |
| 21 | 83740 | H2AFB3 | H2A histone family member B | 3,55 |
| 22 | 23764 | MAFF | v-maf musculoaponeurotic fibrosarcoma oncogene homolog F avian | 3,41 |
| 23 | 1906 | EDN1 | endothelin 1 | 3,40 |
| 24 | 51025 | null | mitochondria-associated protein involved in granulocyte-macrophage colony-stimulating factor signal transduction | 3,11 |
| 25 | 9021 | SOCS3 | suppressor of cytokine signaling 3 | 3,10 |
| 26 | 4790 | NFKB1 | nuclear factor of kappa light polypeptide gene enhancer in B-cells 1 p105 | 3,06 |
| 27 | 22822 | PHLDA1 | pleckstrin homology-like domain family A member 1 | 3,03 |
| 28 | 2615 | LRRC32 | glycoprotein A repetitions predominant | 3,03 |
| 29 | 8659 | ALDH4A1 | aldehyde dehydrogenase 4 family member A1 | 3,02 |
| 30 | 9025 | RNF8 | ring finger protein C3HC4 type 8 | 2,98 |
| 31 | 2152 | F3 | coagulation factor III thromboplastin tissue factor | 2,90 |
| 32 | 10307 | APBB3 | amyloid beta A4 precursor protein-binding family B member 3 | 2,90 |
| 33 | 10560 | SLC19A2 | solute carrier family 19 thiamine transporter member 2 | 2,90 |
| 34 | 25805 | BAMBI | BMP and activin membrane-bound inhibitor homolog Xenopus laevis | 2,88 |
| 35 | 60370 | AVPI1 | arginine vasopressin-induced 1 | 2,87 |
| 36 | 55655 | NALP2 | NACHT leucine rich repeat and PYD containing 2 | 2,83 |
| 37 | 8277 | TKTL1 | transketolase-like 1 | 2,82 |
| 38 | 54847 | SIDT1 | hypothetical protein FLJ20174 | 2,78 |
| 39 | 5138 | PDE2A | phosphodiesterase 2A cGMP-stimulated | 2,77 |
| 40 | 10120 | ACTR1B | ARP1 actin-related protein 1 homolog B centractin beta yeast | 2,70 |
| 41 | 3586 | IL10 | interleukin 10 | 2,69 |
| 42 | 6489 | ST8SIA1 | sialyltransferase 8A alpha-N-acetylneuraminate_ alpha-28-sialyltransferase GD3 synthase | 2,67 |
| 43 | 1960 | EGR3 | early growth response 3 | 2,65 |
| 44 | 10769 | PLK2 | polo-like kinase 2 Drosophila | 2,63 |
| 45 | 1846 | DUSP4 | dual specificity phosphatase 4 | 2,60 |
| 46 | 9076 | CLDN1 | claudin 1 | 2,59 |
| 47 | 51458 | RHCG | Rhesus blood group C glycoprotein | 2,55 |
| 48 | 788 | SLC25A20 | solute carrier family 25 carnitine/acylcarnitine translocase member 20 | 2,55 |
| 49 | 6355 | CCL8 | chemokine C-C motif ligand 8 | 2,54 |
| 50 | 283131 | null | null | 2,53 |
| 51 | 85378 | TUBGCP6 | tubulin gamma complex associated protein 6 | 2,46 |
| 52 | 51365 | PLA1A | phospholipase A1 member A | 2,45 |
| 53 | 6617 | SNAPC1 | small nuclear RNA activating complex polypeptide 1 43kDa | 2,38 |
| 54 | 3202 | HOXA5 | homeo box A5 | 2,37 |
| 55 | 6515 | SLC2A3 | solute carrier family 2 facilitated glucose transporter member 3 | 2,36 |
| 56 | 9363 | RAB33A | RAB33A member RAS oncogene family | 2,36 |
| 57 | 10514 | MYBBP1A | MYB binding protein P160 1a | 2,35 |
| 58 | 5142 | PDE4B | phosphodiesterase 4B cAMP-specific phosphodiesterase E4 dunce homolog Drosophila | 2,35 |
| 59 | 79693 | null | ischemia/reperfusion inducible protein | 2,34 |
| 60 | 983 | CDC2 | cell division cycle 2 G1 to S and G2 to M | 2,31 |
| 61 | 54751 | FBLIM1 | filamin-binding LIM protein-1 | 2,31 |
| 62 | 57823 | SLAMF7 | SLAM family member 7 | 2,30 |
| 63 | 9028 | RHBDL1 | rhomboid veinlet-like 1 Drosophila | 2,28 |
| 64 | 481 | ATP1B1 | ATPase Na+/K+ transporting beta 1 polypeptide | 2,27 |
| 65 | 1647 | GADD45A | growth arrest and DNA-damage-inducible alpha | 2,23 |
| 66 | 6004 | RGS16 | regulator of G-protein signalling 16 | 2,22 |
| 67 | 64108 | null | 28kD interferon responsive protein | 2,22 |
| 68 | 83660 | TLN2 | talin 2 | 2,21 |
| 69 | 2322 | FLT3 | fms-related tyrosine kinase 3 | 2,20 |
| 70 | 5894 | RAF1 | v-raf-1 murine leukemia viral oncogene homolog 1 | 2,19 |
| 71 | 7056 | THBD | thrombomodulin | 2,15 |
| 72 | 1939 | LGTN | ligatin | 2,15 |
| 73 | 29970 | SCHIP1 | schwannomin interacting protein 1 | 2,15 |
| 74 | 57801 | HES4 | bHLH factor Hes4 | 2,13 |
| 75 | 23529 | CLCF1 | cardiotrophin-like cytokine | 2,12 |
| 76 | 23135 | JMJD3 | jumonji domain containing 3 | 2,11 |
| 77 | 1164 | CKS2 | CDC28 protein kinase regulatory subunit 2 | 2,11 |
| 78 | 7003 | TEAD1 | TEA domain family member 1 SV40 transcriptional enhancer factor | 2,11 |
| 79 | 7422 | VEGF | vascular endothelial growth factor | 2,10 |
| 80 | 9590 | AKAP12 | A kinase PRKA anchor protein gravin 12 | 2,09 |
| 81 | 27285 | TEKT2 | tektin 2 testicular | 2,08 |
| 82 | 7128 | TNFAIP3 | tumor necrosis factor alpha-induced protein 3 | 2,06 |
| 83 | 3697 | ITIH1 | inter-alpha globulin inhibitor H1 | 2,05 |
| 84 | 4216 | MAP3K4 | mitogen-activated protein kinase kinase kinase 4 | 2,05 |
| 85 | 3280 | HES1 | hairy and enhancer of split 1 Drosophila | 2,02 |
| 86 | 1440 | CSF3 | colony stimulating factor 3 granulocyte | 2,02 |
| 87 | 114112 | TXNRD3 | thioredoxin reductase 3 | 2,01 |
| 88 | 11182 | SLC2A6 | solute carrier family 2 facilitated glucose transporter member 6 | 2,00 |
| 89 | 7351 | UCP2 | uncoupling protein 2 mitochondrial proton carrier | 2,00 |
| 90 | 8364 | HIST1H4C | histone 1 H4c | 1,99 |
| 91 | 4209 | MEF2D | MADS box transcription enhancer factor 2 polypeptide D myocyte enhancer factor 2D | 1,94 |
| 92 | 8811 | GALR2 | galanin receptor 2 | 1,94 |
| 93 | 5968 | REG1B | regenerating islet-derived 1 beta pancreatic stone protein pancreatic thread protein | 1,86 |
| 94 | 23187 | PHLDB1 | pleckstrin homology-like domain family B member 1 | 1,86 |
| 95 | 136 | ADORA2B | adenosine A2b receptor | 1,72 |
| 96 | 10365 | KLF2 | Kruppel-like factor 2 lung | 1,71 |
| 97 | 6366 | CCL21 | chemokine C-C motif ligand 21 | 1,71 |
| 98 | 6696 | SPP1 | secreted phosphoprotein 1 osteopontin bone sialoprotein I early T-lymphocyte activation 1 | 1,70 |
| 99 | 64090 | GAL3ST2 | galactose-3-O-sulfotransferase 2 | 1,69 |
| 100 | 1052 | CEBPD | CCAAT/enhancer binding protein C/EBP delta | 1,64 |
| 101 | 8740 | TNFSF14 | tumor necrosis factor ligand superfamily member 14 | 1,60 |
| 102 | 9955 | HS3ST3A1 | heparan sulfate glucosamine 3-O-sulfotransferase 3A1 | 1,18 |
