# Supplementary material for: Gram-positive pathogenic bacteria induce a common early response in human monocytes
Source: BMC Microbiol. 2010 Nov 2;10:275. doi: 10.1186/1471-2180-10-275 (PMC2988769; doi:10.1186/1471-2180-10-275)
Supplement: Additional file 6 — Table S6. S. pneumoniae - Totally downregulated genes. FDR 10 [file 1471-2180-10-275-S6.DOC]

**Table S6.** *S. pneumoniae –* Totally downregulated genes. FDR 10.

| **No.** | **Gene IDs** | **Gene Symbol** | **Gene Name** | **Fold Change** |
| --- | --- | --- | --- | --- |
| 1 | 26224 | FBXL3 | F-box and leucine-rich repeat protein 3 | -3,01 |
| 2 | 4064 | CD180 | lymphocyte antigen 64 homolog radioprotective 105kDa mouse | -3,00 |
| 3 | 54554 | WDR5B | WD repeat domain 5B | -2,98 |
| 4 | 23335 | WDR7 | WD repeat domain 7 | -2,74 |
| 5 | 92342 | null | hypothetical protein MGC9084 | -2,74 |
| 6 | 80818 | ZNF436 | zinc finger protein 436 | -2,72 |
| 7 | 132241 | null | hypothetical protein LOC132241 | -2,67 |
| 8 | 51530 | ZC3HC1 | nuclear interacting partner of anaplastic lymphoma kinase ALK | -2,64 |
| 9 | 148479 | PHF13 | PHD finger protein 13 | -2,59 |
| 10 | 10978 | null | ATP/GTP-binding protein | -2,59 |
| 11 | 55794 | DDX28 | DEAD Asp-Glu-Ala-Asp box polypeptide 28 | -2,56 |
| 12 | 9529 | BAG5 | BCL2-associated athanogene 5 | -2,56 |
| 13 | 7096 | TLR1 | toll-like receptor 1 | -2,53 |
| 14 | 901 | CCNG2 | cyclin G2 | -2,53 |
| 15 | 11146 | GLMN | glomulin FKBP associated protein | -2,49 |
| 16 | 8799 | PEX11B | peroxisomal biogenesis factor 11B | -2,44 |
| 17 | 63915 | MUTED | muted homolog mouse | -2,42 |
| 18 | 7568 | ZNF20 | zinc finger protein 20 KOX 13 | -2,41 |
| 19 | 79891 | ZNF671 | hypothetical protein FLJ23506 | -2,39 |
| 20 | 57567 | ZNF319 | zinc finger protein 319 | -2,36 |
| 21 | 57561 | ARRDC3 | arrestin domain containing 3 | -2,36 |
| 22 | 10116 | FEM1B | fem-1 homolog b C. elegans | -2,34 |
| 23 | 26127 | FGFR1OP2 | FGFR1 oncogene partner 2 | -2,33 |
| 24 | 9655 | SOCS5 | suppressor of cytokine signaling 5 | -2,33 |
| 25 | 51126 | NAT5 | N-acetyltransferase 5 ARD1 homolog S. cerevisiae | -2,31 |
| 26 | 7728 | ZNF175 | zinc finger protein 175 | -2,27 |
| 27 | 57547 | ZNF624 | zinc finger protein 624 | -2,26 |
| 28 | 1050 | CEBPA | CCAAT/enhancer binding protein C/EBP alpha | -2,19 |
| 29 | 7559 | null | null | -2,18 |
| 30 | 8772 | FADD | Fas TNFRSF6-associated via death domain | -2,13 |
| 31 | 10773 | ZNF482 | zinc finger protein 482 | -2,11 |
| 32 | 91574 | null | hypothetical protein FLJ38663 | -2,04 |
| 33 | 51058 | ZNF691 | hypothetical protein LOC51058 | -2,01 |
| 34 | 874 | CBR3 | carbonyl reductase 3 | -2,01 |
| 35 | 55330 | CNO | cappuccino | -1,96 |
| 36 | 5718 | PSMD12 | proteasome prosome macropain 26S subunit non-ATPase 12 | -1,95 |
| 37 | 10668 | CGRRF1 | cell growth regulator with ring finger domain 1 | -1,92 |
| 38 | 8629 | JRK | jerky homolog mouse | -1,84 |
