# Supplementary material for: Gram-positive pathogenic bacteria induce a common early response in human monocytes
Source: BMC Microbiol. 2010 Nov 2;10:275. doi: 10.1186/1471-2180-10-275 (PMC2988769; doi:10.1186/1471-2180-10-275)
Supplement: Additional file 7 — Table S7. L. monocytogenes - Specifically upregulated genes. FDR 10 [file 1471-2180-10-275-S7.DOC]

**Table S7.** *L. monocytogenes –* Specifically upregulated genes. FDR 10.

| **No.** | **Gene IDs** | **Gene Symbol** | **Gene Name** | **Fold Change** |
| --- | --- | --- | --- | --- |
| 1 | 4853 | NOTCH2NL | Notch homolog 2 (Drosophila) N-terminal like | 3,4611 |
| 2 | 11065 | UBE2C | Ubiquitin-conjugating enzyme E2C | 2,7979 |
| 3 | 196441 | MGC23401 | Proline/serine-rich coiled-coil 2 | 2,5299 |
| 4 | 55771 | FLJ11029 | Proline rich 11 | 2,4480 |
| 5 | 79073 | MGC5508 | Hypothetical protein MGC5508 | 2,4249 |
| 6 | 6929 | TCF3 | Transcription factor 3 (E2A immunoglobulin enhancer binding factors E12/E47) | 2,3730 |
| 7 | 5277 | PIGA | Phosphatidylinositol glycan, class A (paroxysmal nocturnal hemoglobinuria)"" | 2,3670 |
| 8 | 127435 | PODN | Podocan | 2,3117 |
| 9 | 1827 | DSCR1 | Down syndrome critical region gene 1 | 2,2824 |
| 10 | 1277 | COL1A1 | Collagen, type I, alpha 1"" | 2,2715 |
| 11 | 26051 | PPP1R16B | Protein phosphatase 1, regulatory (inhibitor) subunit 16B"" | 2,2570 |
| 12 | 5266 | PI3 | Peptidase inhibitor 3, skin-derived (SKALP)"" | 2,2381 |
| 13 | 3572 | IL6ST | Interleukin 6 signal transducer (gp130, oncostatin M receptor)"" | 2,2037 |
| 14 | 8756 | ADAM7 | ADAM metallopeptidase domain 7 | 2,1864 |
| 15 | 51186 | WBP5 | WW domain binding protein 5 | 2,1785 |
| 16 | 844 | CASQ1 | Calsequestrin 1 (fast-twitch, skeletal muscle)"" | 2,1090 |
| 17 | 9026 | HIP1R | Huntingtin interacting protein-1-related | 2,1085 |
| 18 | 2623 | GATA1 | GATA binding protein 1 (globin transcription factor 1) | 2,1026 |
| 19 | 55228 | FLJ10781 | Hypothetical protein FLJ10781 | 2,0741 |
| 20 | 10505 | SEMA4F | Sema domain, immunoglobulin domain (Ig), transmembrane domain (TM) and short cytoplasmic domain, (semaphorin) 4F"" | 2,0656 |
| 21 | 30819 | KCNIP2 | Kv channel interacting protein 2 | 2,0635 |
| 22 | 26256 | CABYR | Calcium binding tyrosine-(Y)-phosphorylation regulated (fibrousheathin 2) | 2,0490 |
| 23 | 9256 | BZRAP1 | Benzodiazapine receptor (peripheral) associated protein 1 | 2,0384 |
| 24 | 55633 | TBC1D22B | TBC1 domain family, member 22B"" | 2,0359 |
| 25 | 3158 | HMGCS2 | 3-hydroxy-3-methylglutaryl-Coenzyme A synthase 2 (mitochondrial) | 2,0312 |
| 26 | 6288 | SAA1 | Serum amyloid A2 | 2,0288 |
| 27 | 27289 | RND1 | Rho family GTPase 1 | 2,0206 |
| 28 | 7466 | WFS1 | Wolfram syndrome 1 (wolframin) | 2,0140 |
| 29 | 23099 | ZNF297B | Zinc finger protein 297B | 1,9997 |
| 30 | 1875 | E2F5 | E2F transcription factor 5, p130-binding"" | 1,9990 |
| 31 | 7062 | THH | Trichohyalin | 1,9971 |
| 32 | 3777 | KCNK3 | Potassium channel, subfamily K, member 3"" | 1,9903 |
| 33 | 9221 | NOLC1 | Nucleolar and coiled-body phosphoprotein 1 | 1,9758 |
| 34 | 8424 | BBOX1 | Butyrobetaine (gamma), 2-oxoglutarate dioxygenase (gamma-butyrobetaine hydroxylase) 1"" | 1,9727 |
| 35 | 5989 | RFX1 | Regulatory factor X, 1 (influences HLA class II expression)"" | 1,9648 |
| 36 | 4293 | MAP3K9 | Mitogen-activated protein kinase kinase kinase 9 | 1,9617 |
| 37 | 6947 | TCN1 | Transcobalamin I (vitamin B12 binding protein, R binder family)"" | 1,9613 |
| 38 | 55311 | ZNF444 | Zinc finger protein 444 | 1,9598 |
| 39 | 6692 | SPINT1 | Serine peptidase inhibitor, Kunitz type 1"" | 1,9562 |
| 40 | 22930 | RAB3GAP | RAB3 GTPase activating protein subunit 1 (catalytic) | 1,9495 |
| 41 | 4288 | MKI67 | Antigen identified by monoclonal antibody Ki-67 | 1,9380 |
| 42 | 4603 | MYBL1 | V-myb myeloblastosis viral oncogene homolog (avian)-like 1 | 1,9373 |
| 43 | 22869 | ZNF510 | Zinc finger protein 510 | 1,9278 |
| 44 | 50515 | CHST11 | Carbohydrate (chondroitin 4) sulfotransferase 11 | 1,9248 |
| 45 | 10308 | ZNF267 | Zinc finger protein 267 | 1,9096 |
| 46 | 9262 | STK17B | Serine/threonine kinase 17b (apoptosis-inducing) | 1,9087 |
| 47 | 5805 | PTS | 6-pyruvoyltetrahydropterin synthase | 1,9049 |
| 48 | 1837 | DTNA | Dystrobrevin, alpha"" | 1,9028 |
| 49 | 2204 | FCAR | Fc fragment of IgA, receptor for"" | 1,8936 |
| 50 | 11096 | ADAMTS5 | ADAM metallopeptidase with thrombospondin type 1 motif, 5 (aggrecanase-2)"" | 1,8881 |
| 51 | 10396 | ATP8A1 | ATPase, aminophospholipid transporter (APLT), Class I, type 8A, member 1"" | 1,5467 |
