# Supplementary material for: Gram-positive pathogenic bacteria induce a common early response in human monocytes
Source: BMC Microbiol. 2010 Nov 2;10:275. doi: 10.1186/1471-2180-10-275 (PMC2988769; doi:10.1186/1471-2180-10-275)
Supplement: Additional file 8 — Table S8. L. monocytogenes - Specifically downregulated genes. FDR 10 [file 1471-2180-10-275-S8.DOC]

**Table S8.** *L. monocytogenes* – Specifically downregulated genes. FDR 10

| **No.** | **Gene IDs** | **Gene Symbol** | **Gene Name** | **Fold Change** |
| --- | --- | --- | --- | --- |
| 1 | 10520 | ZNF211 | Zinc finger protein 211 | -3,16 |
| 2 | 5511 | PPP1R8 | Protein phosphatase 1, regulatory (inhibitor) subunit 8"" | -3,02 |
| 3 | 8509 | NDST2 | N-deacetylase/N-sulfotransferase (heparan glucosaminyl) 2 | -2,92 |
| 4 | 8437 | RASAL1 | RAS protein activator like 1 (GAP1 like) | -2,88 |
| 5 | 22928 | SEPHS2 | Selenophosphate synthetase 2 | -2,77 |
| 6 | 7270 | TTF1 | Transcription termination factor, RNA polymerase I"" | -2,77 |
| 7 | 7596 | ZNF45 | Zinc finger protein 45 | -2,76 |
| 8 | 2805 | GOT1 | Glutamic-oxaloacetic transaminase 1, soluble (aspartate aminotransferase 1)"" | -2,66 |
| 9 | 10522 | DEAF1 | Deformed epidermal autoregulatory factor 1 (Drosophila) | -2,60 |
| 10 | 10885 | WDR3 | WD repeat domain 3 | -2,55 |
| 11 | 50615 | IL21R | Interleukin 21 receptor | -2,54 |
| 12 | 10845 | CLPX | ClpX caseinolytic peptidase X homolog (E. coli) | -2,51 |
| 13 | 9938 | ARHGAP25 | Rho GTPase activating protein 25 | -2,43 |
| 14 | 150290 | DUSP18 | Dual specificity phosphatase 18 | -2,41 |
| 15 | 7737 | ZNF183 | Ring finger protein 113A | -2,41 |
| 16 | 11244 | ZHX1 | Zinc fingers and homeoboxes 1 | -2,40 |
| 17 | 26269 | FBXO8 | F-box protein 8 | -2,39 |
| 18 | 23300 | MGC47816 | Family with sequence similarity 80, member A"" | -2,39 |
| 19 | 79102 | RNF26 | Ring finger protein 26 | -2,39 |
| 20 | 7570 | ZNF22 | Zinc finger protein 22 (KOX 15) | -2,38 |
| 21 | 5279 | PIGC | Phosphatidylinositol glycan, class C"" | -2,38 |
| 22 | 4247 | MGAT2 | Mannosyl (alpha-1,6-)-glycoprotein beta-1,2-N-acetylglucosaminyltransferase"" | -2,36 |
| 23 | 23580 | CDC42EP4 | CDC42 effector protein (Rho GTPase binding) 4 | -2,35 |
| 24 | 81488 | GRINL1A | Glutamate receptor, ionotropic, N-methyl D-aspartate-like 1A"" | -2,35 |
| 25 | 56342 | PPAN | Peter pan homolog (Drosophila) | -2,34 |
| 26 | 11334 | TUSC2 | Tumor suppressor candidate 2 | -2,34 |
| 27 | 29914 | TERE1 | UbiA prenyltransferase domain containing 1 | -2,33 |
| 28 | 54826 | FLJ20125 | Hypothetical protein FLJ20125 | -2,30 |
| 29 | 55153 | SDAD1 | SDA1 domain containing 1 | -2,30 |
| 30 | 5031 | P2RY6 | Pyrimidinergic receptor P2Y, G-protein coupled, 6"" | -2,30 |
| 31 | 8846 | ALKBH | AlkB, alkylation repair homolog (E. coli)"" | -2,30 |
| 32 | 7268 | TTC4 | Tetratricopeptide repeat domain 4 | -2,29 |
| 33 | 669 | BPGM | 2,3-bisphosphoglycerate mutase"" | -2,28 |
| 34 | 1052 | CEBPD | CCAAT/enhancer binding protein (C/EBP), delta"" | -2,27 |
| 35 | 7764 | ZNF217 | Zinc finger protein 217 | -2,25 |
| 36 | 7132 | TNFRSF1A | Tumor necrosis factor receptor superfamily, member 1A"" | -2,23 |
| 37 | 5977 | DPF2 | D4, zinc and double PHD fingers family 2"" | -2,21 |
| 38 | 6513 | SLC2A1 | Solute carrier family 2 (facilitated glucose transporter), member 1"" | -2,20 |
| 39 | 55339 | WDR33 | WD repeat domain 33 | -2,20 |
| 40 | 11266 | DUSP12 | Dual specificity phosphatase 12 | -2,20 |
| 41 | 53838 | C11orf24 | Chromosome 11 open reading frame 24 | -2,19 |
| 42 | 7398 | USP1 | Ubiquitin specific peptidase 1 | -2,18 |
| 43 | 54861 | SNRK | SNF related kinase | -2,17 |
| 44 | 1974 | EIF4A2 | Eukaryotic translation initiation factor 4A, isoform 2"" | -2,17 |
| 45 | 10128 | LRPPRC | Leucine-rich PPR-motif containing | -2,16 |
| 46 | 51523 | RPS2 | RNA, U64 small nucleolar"" | -2,16 |
| 47 | 6195 | RPS6KA1 | Ribosomal protein S6 kinase, 90kDa, polypeptide 1"" | -2,16 |
| 48 | 9534 | ZNF539 | Zinc finger protein 254 | -2,16 |
| 49 | 7343 | UBTF | Upstream binding transcription factor, RNA polymerase I"" | -2,15 |
| 50 | 23212 | RRS1 | RRS1 ribosome biogenesis regulator homolog (S. cerevisiae) | -2,14 |
| 51 | 7884 | SLBP | Stem-loop (histone) binding protein | -2,12 |
| 52 | 5929 | RBBP5 | Retinoblastoma binding protein 5 | -2,12 |
| 53 | 60496 | AASDHPPT | Aminoadipate-semialdehyde dehydrogenase-phosphopantetheinyl transferase | -2,12 |
| 54 | 26168 | SENP3 | SUMO1/sentrin/SMT3 specific peptidase 3 | -2,12 |
| 55 | 4690 | NCK1 | NCK adaptor protein 1 | -2,12 |
| 56 | 10458 | BAIAP2 | BAI1-associated protein 2 | -2,09 |
| 57 | 55030 | FBXO34 | F-box protein 34 | -2,09 |
| 58 | 54851 | FGIF | Ankyrin repeat domain 49 | -2,06 |
| 59 | 11108 | PRDM4 | PR domain containing 4 | -2,06 |
| 60 | 55602 | CARF | Collaborates/cooperates with ARF (alternate reading frame) protein | -2,06 |
| 61 | 400657 | LOC400657 | Hypothetical gene supported by BC036588 | -2,05 |
| 62 | 11277 | TREX1 | Three prime repair exonuclease 1 | -2,05 |
| 63 | 6049 | RNF6 | Ring finger protein (C3H2C3 type) 6 | -2,05 |
| 64 | 53918 | PELO | Pelota homolog (Drosophila) | -2,03 |
| 65 | 5134 | PDCD2 | Programmed cell death 2 | -2,03 |
| 66 | 51244 | C3orf19 | Chromosome 3 open reading frame 19 | -2,03 |
| 67 | 8131 | C16orf35 | Chromosome 16 open reading frame 35 | -2,03 |
| 68 | 10884 | MRPS30 | Mitochondrial ribosomal protein S30 | -2,02 |
| 69 | 10693 | CCT6B | Chaperonin containing TCP1, subunit 6B (zeta 2)"" | -2,02 |
| 70 | 55182 | FLJ10597 | Chromosome 1 open reading frame 164 | -2,02 |
| 71 | 10044 | SH2D3C | SH2 domain containing 3C | -2,02 |
| 72 | 10322 | SMYD5 | SMYD family member 5 | -2,01 |
| 73 | 10084 | PQBP1 | Polyglutamine binding protein 1 | -2,01 |
| 74 | 64785 | FLJ13912 | Hypothetical protein FLJ13912 | -2,00 |
| 75 | 9889 | ZBED4 | Zinc finger, BED-type containing 4"" | -2,00 |
| 76 | 9183 | ZW10 | ZW10, kinetochore associated, homolog (Drosophila)"" | -1,99 |
| 77 | 80019 | UBTD1 | Ubiquitin domain containing 1 | -1,99 |
| 78 | 57542 | KIAA1340 | Kelch domain containing 5 | -1,99 |
| 79 | 10795 | ZNF268 | Zinc finger protein 268 | -1,98 |
| 80 | 10023 | FRAT1 | Frequently rearranged in advanced T-cell lymphomas | -1,97 |
| 81 | 8364 | HIST1H4C | Histone 1, H4c"" | -1,97 |
| 82 | 7358 | UGDH | UDP-glucose dehydrogenase | -1,97 |
| 83 | 10626 | TRIM16 | Tripartite motif-containing 16 | -1,97 |
| 84 | 2214 | FCGR3A | Fc fragment of IgG, low affinity IIIa, receptor (CD16a)"" | -1,96 |
| 85 | 56985 | MDS006 | Chromosome 17 open reading frame 48 | -1,96 |
| 86 | 333926 | PPP2CZ | Protein phosphatase 1J (PP2C domain containing) | -1,96 |
| 87 | 11124 | FAF1 | Fas (TNFRSF6) associated factor 1 | -1,95 |
| 88 | 9046 | DOK2 | Docking protein 2, 56kDa"" | -1,95 |
| 89 | 55905 | ZNF313 | Zinc finger protein 313 | -1,94 |
| 90 | 10215 | OLIG2 | Oligodendrocyte lineage transcription factor 2 | -1,91 |
| 91 | 57569 | ARHGAP20 | Rho GTPase activating protein 20 | -1,88 |
| 92 | 55658 | RNF126 | Ring finger protein 126 | -1,75 |
| 93 | 57479 | KIAA1205 | KIAA1205 | -1,73 |
| 94 | 7436 | VLDLR | Very low density lipoprotein receptor | -1,24 |
| 95 | 171023 | ASXL1 | Additional sex combs like 1 (Drosophila) | -1,35 |
