# Supplementary material for: Gram-positive pathogenic bacteria induce a common early response in human monocytes
Source: BMC Microbiol. 2010 Nov 2;10:275. doi: 10.1186/1471-2180-10-275 (PMC2988769; doi:10.1186/1471-2180-10-275)
Supplement: Additional file 9 — Table S9. S. aureus - Specifically upregulated genes. FDR 10 [file 1471-2180-10-275-S9.DOC]

**Table S9.** *S. aureus* - Specifically upregulated genes. FDR 10

| **No.** | **Gene IDs** | **Gene Symbol** | **Gene Name** | **Fold Change** |
| --- | --- | --- | --- | --- |
| 1 | 11080 | DNAJB4 | DnaJ (Hsp40) homolog, subfamily B, member 4"" | 3,12 |
| 2 | 8874 | ARHGEF7 | Rho guanine nucleotide exchange factor (GEF) 7 | 2,90 |
| 3 | 80176 | SSB1 | SplA/ryanodine receptor domain and SOCS box containing 1 | 2,63 |
| 4 | 8553 | BHLHB2 | Basic helix-loop-helix domain containing, class B, 2"" | 2,60 |
| 5 | 23306 | KIAA0286 | KIAA0286 protein | 2,57 |
| 6 | 2956 | MSH6 | MutS homolog 6 (E. coli) | 2,53 |
| 7 | 1594 | CYP27B1 | Cytochrome P450, family 27, subfamily B, polypeptide 1"" | 2,49 |
| 8 | 27076 | C4.4A | LY6/PLAUR domain containing 3 | 2,46 |
| 9 | 2703 | GJA8 | Gap junction protein, alpha 8, 50kDa (connexin 50)"" | 2,39 |
| 10 | 7159 | TP53BP2 | Tumor protein p53 binding protein, 2"" | 2,38 |
| 11 | 51299 | NRN1 | Neuritin 1 | 2,36 |
| 12 | 182 | JAG1 | Jagged 1 (Alagille syndrome) | 2,36 |
| 13 | 5155 | PDGFB | Platelet-derived growth factor beta polypeptide (simian sarcoma viral (v-sis) oncogene homolog) | 2,32 |
| 14 | 6133 | RPL9 | Ribosomal protein L9 | 2,28 |
| 15 | 958 | CD40 | CD40 antigen (TNF receptor superfamily member 5) | 2,25 |
| 16 | 29113 | C6orf15 | Chromosome 6 open reading frame 15 | 2,24 |
| 17 | 149830 | LOC149830 | Prion protein (testis specific) | 2,22 |
| 18 | 1009 | CDH11 | Cadherin 11, type 2, OB-cadherin (osteoblast)"" | 2,20 |
| 19 | 57570 | KIAA1393 | TRM5 tRNA methyltransferase 5 homolog (S. cerevisiae) | 2,20 |
| 20 | 4157 | MC1R | Melanocortin 1 receptor (alpha melanocyte stimulating hormone receptor) | 2,16 |
| 21 | 2842 | GPR19 | G protein-coupled receptor 19 | 2,15 |
| 22 | 340371 | NRBP2 | Nuclear receptor binding protein 2 | 2,15 |
| 23 | 5272 | SERPINB9 | Serpin peptidase inhibitor, clade B (ovalbumin), member 9"" | 2,12 |
| 24 | 329 | BIRC2 | Baculoviral IAP repeat-containing 2 | 2,10 |
| 25 | 3601 | IL15RA | Interleukin 15 receptor, alpha"" | 2,10 |
| 26 | 3680 | ITGA9 | Integrin, alpha 9"" | 2,09 |
| 27 | 10998 | FLJ20626 | Zinc finger protein 446 | 2,09 |
| 28 | 9576 | SPAG6 | Sperm associated antigen 6 | 2,09 |
| 29 | 10370 | CITED2 | Cbp/p300-interacting transactivator, with Glu/Asp-rich carboxy-terminal domain, 2"" | 2,08 |
| 30 | 7706 | TRIM25 | Tripartite motif-containing 25 | 2,08 |
| 31 | 10974 | C10orf116 | Chromosome 10 open reading frame 116 | 2,07 |
| 32 | 51703 | EIF3S3 | Eukaryotic translation initiation factor 3, subunit 3 gamma, 40kDa"" | 2,07 |
| 33 | 389337 | FLJ41603 | FLJ41603 protein | 2,06 |
| 34 | 1672 | DEFB1 | Defensin, beta 1"" | 2,06 |
| 35 | 27342 | RABGEF1 | RAB guanine nucleotide exchange factor (GEF) 1 | 2,06 |
| 36 | 1948 | EFNB2 | Ephrin-B2 | 2,04 |
| 37 | 58476 | TP53INP2 | Tumor protein p53 inducible nuclear protein 2 | 2,00 |
| 38 | 3068 | HDGF | Hepatoma-derived growth factor (high-mobility group protein 1-like) | 1,98 |
| 39 | 23189 | ANKRD15 | Ankyrin repeat domain 15 | 1,94 |
