# Supplementary material for: Gram-positive pathogenic bacteria induce a common early response in human monocytes
Source: BMC Microbiol. 2010 Nov 2;10:275. doi: 10.1186/1471-2180-10-275 (PMC2988769; doi:10.1186/1471-2180-10-275)
Supplement: Additional file 10 — Table S10. S. aureus - Specifically downregulated genes. FDR 10 [file 1471-2180-10-275-S10.DOC]

**Table S10.** *S. aureus* – Specifically downregulated genes. FDR 10.

| **No.** | **Gene IDs** | **Gene Symbol** | **Gene Name** | **Fold Change** |
| --- | --- | --- | --- | --- |
| 1 | 51275 | FLJ39616 | Apoptosis-related protein PNAS-1 | -2,87 |
| 2 | 5698 | PSMB9 | Proteasome (prosome, macropain) subunit, beta type, 9 (large multifunctional peptidase 2)"" | -2,81 |
| 3 | 25949 | ZNF501 | Zinc finger protein 501 | -2,70 |
| 4 | 11018 | TMED1 | Transmembrane emp24 protein transport domain containing 1 | -2,56 |
| 5 | 130916 | SNED1 | Sushi, nidogen and EGF-like domains 1"" | -2,48 |
| 6 | 29100 | HSPC171 | HSPC171 protein | -2,44 |
| 7 | 2992 | GYG | Glycogenin 1 | -2,42 |
| 8 | 55272 | C15orf12 | IMP3, U3 small nucleolar ribonucleoprotein, homolog (yeast)"" | -2,41 |
| 9 | 6341 | SCO1 | SCO cytochrome oxidase deficient homolog 1 (yeast) | -2,41 |
| 10 | 9412 | SURB7 | SRB7 suppressor of RNA polymerase B homolog (yeast) | -2,29 |
| 11 | 90407 | TMEM41A | Transmembrane protein 41A | -2,23 |
| 12 | 7905 | C5orf18 | Chromosome 5 open reading frame 18 | -2,20 |
| 13 | 25940 | DKFZP564F0522 | Family with sequence similarity 98, member A"" | -2,19 |
| 14 | 11344 | PTK9L | PTK9L protein tyrosine kinase 9-like (A6-related protein) | -2,19 |
| 15 | 9587 | MAD2L1BP | MAD2L1 binding protein | -2,17 |
| 16 | 26098 | C10orf137 | Chromosome 10 open reading frame 137 | -2,15 |
| 17 | 81689 | HBLD2 | HESB like domain containing 2 | -2,12 |
| 18 | 84337 | MGC4549 | Elongation factor 1 homolog (ELF1, S. cerevisiae)"" | -2,10 |
| 19 | 51023 | MRPS18C | Mitochondrial ribosomal protein S18C | -2,08 |
| 20 | 1477 | CSTF1 | Cleavage stimulation factor, 3' pre-RNA, subunit 1, 50kDa"" | -2,08 |
| 21 | 6941 | TCF19 | Transcription factor 19 (SC1) | -2,06 |
| 22 | 80778 | ZNF34 | Zinc finger protein 34 (KOX 32) | -2,06 |
| 23 | 57150 | C6orf162 | Chromosome 6 open reading frame 164 | -2,04 |
| 24 | 8976 | WASL | Wiskott-Aldrich syndrome-like | -2,03 |
| 25 | 6118 | RPA2 | Replication protein A2, 32kDa"" | -2,02 |
| 26 | 26157 | GIMAP2 | GTPase, IMAP family member 2"" | -2,01 |
| 27 | 27440 | CECR5 | Cat eye syndrome chromosome region, candidate 5"" | -2,01 |
| 28 | 10102 | TSFM | Ts translation elongation factor, mitochondrial"" | -1,99 |
| 29 | 51106 | TFB1M | Transcription factor B1, mitochondrial"" | -1,98 |
| 30 | 57864 | TSCOT | Thymic stromal co-transporter | -1,88 |
| 31 | 974 | CD79B | CD79B antigen (immunoglobulin-associated beta) | -1,86 |
| 32 | 55303 | GIMAP4 | GTPase, IMAP family member 4"" | -1,80 |
| 33 | 114882 | OSBPL8 | Oxysterol binding protein-like 8 | -1,69 |
| 34 | 9167 | COX7A2L | Cytochrome c oxidase subunit VIIa polypeptide 2 like | -1,43 |
