# Supplementary material for: Gram-positive pathogenic bacteria induce a common early response in human monocytes
Source: BMC Microbiol. 2010 Nov 2;10:275. doi: 10.1186/1471-2180-10-275 (PMC2988769; doi:10.1186/1471-2180-10-275)
Supplement: Additional file 11 — Table S11. S. pneumoniae - Specifically upregulated genes. FDR 10 [file 1471-2180-10-275-S11.DOC]

**Table S11.** *S. pneumoniae* – Specifically upregulated genes. FDR 10.

| **No.** | **Gene IDs** | **Gene Symbol** | **Gene Name** | **Fold Change** |
| --- | --- | --- | --- | --- |
| 1 | 3624 | INHBA | Inhibin, beta A (activin A, activin AB alpha polypeptide)"" | 8,55 |
| 2 | 8013 | NR4A3 | Nuclear receptor subfamily 4, group A, member 3"" | 8,40 |
| 3 | 23764 | MAFF | V-maf musculoaponeurotic fibrosarcoma oncogene homolog F (avian) | 3,41 |
| 4 | 60370 | AVPI1 | Arginine vasopressin-induced 1 | 2,87 |
| 5 | 5138 | PDE2A | Phosphodiesterase 2A, cGMP-stimulated"" | 2,77 |
| 6 | 3586 | IL10 | Interleukin 10 | 2,69 |
| 7 | 10769 | PLK2 | Polo-like kinase 2 (Drosophila) | 2,63 |
| 8 | 79693 | FLJ23476 | YrdC domain containing (E.coli) | 2,34 |
| 9 | 54751 | FBLP-1 | Filamin binding LIM protein 1 | 2,31 |
| 10 | 1647 | GADD45A | Growth arrest and DNA-damage-inducible, alpha"" | 2,23 |
| 11 | 29970 | SCHIP1 | Schwannomin interacting protein 1 | 2,15 |
| 12 | 23135 | JMJD3 | Jumonji domain containing 3 | 2,11 |
| 13 | 1440 | CSF3 | Colony stimulating factor 3 (granulocyte) | 2,02 |
| 14 | 7351 | UCP2 | Uncoupling protein 2 (mitochondrial, proton carrier)"" | 2,00 |
| 15 | 8364 | HIST1H4C | Histone 1, H4c"" | 1,99 |
| 16 | 4209 | MEF2D | MADS box transcription enhancer factor 2, polypeptide D (myocyte enhancer factor 2D)"" | 1,94 |
| 17 | 10365 | KLF2 | Kruppel-like factor 2 (lung) | 1,71 |
| 18 | 6366 | CCL21 | Chemokine (C-C motif) ligand 21 | 1,71 |
| 19 | 64090 | GAL3ST2 | Galactose-3-O-sulfotransferase 2 | 1,69 |
| 20 | 1052 | CEBPD | CCAAT/enhancer binding protein (C/EBP), delta"" | 1,64 |
