# Supplementary material for: Gram-positive pathogenic bacteria induce a common early response in human monocytes
Source: BMC Microbiol. 2010 Nov 2;10:275. doi: 10.1186/1471-2180-10-275 (PMC2988769; doi:10.1186/1471-2180-10-275)
Supplement: Additional file 12 — Table S12. S. pneumoniae - Specifically downregulated genes. FDR 10 [file 1471-2180-10-275-S12.DOC]

**Table S12.** *S. pneumoniae* – Specifically downregulated genes. FDR 10.

| **No.** | **Gene IDs** | **Gene Symbol** | **Gene Name** | **Fold Change** |
| --- | --- | --- | --- | --- |
| 1 | 23335 | WDR7 | WD repeat domain 7 | -2,74 |
| 2 | 11146 | GLMN | Glomulin, FKBP associated protein"" | -2,49 |
