# Supplementary material for: Gram-positive pathogenic bacteria induce a common early response in human monocytes
Source: BMC Microbiol. 2010 Nov 2;10:275. doi: 10.1186/1471-2180-10-275 (PMC2988769; doi:10.1186/1471-2180-10-275)
Supplement: Additional file 15 — Figure S1. Correlation of Fold Change. Relative expression of 14 genes as determined by real time RT-PCR upon infection plotted against their corresponding microarray values. Results are averaged for all 5 donors. [file 1471-2180-10-275-S15.DOC]

**Table S13. Relative quantification of IL12A, IL12B/IL23B, IL23A and IFNγ by real time RT-PCR.** Relative expression of IL12A, IL12B/IL23B, IL23A and IFNγ (IFNG) mRNAs as determined by real time RT-PCR. Results are Fold Change values of infected vs. noninfected cells. LM: *L. monocytogenes* EGDe, SA: *S. aureus*, SP: *S. pneumoniae,* AM: arithmetical mean, SD: standard deviation.

| D20 | LM | **SA** | **SP** |
| --- | --- | --- | --- |
| IL12A | 1,08 | -1,42 | -1,75 |
| IL12B/IL23B | 3,14 | 30,13 | 22,83 |
| IL23A | 9,03 | 8,49 | 2,45 |
| IFNG | 1,77 | -1,05 | -3,33 |
| **D21** | **LM** | **SA** | **SP** |
| IL12A | 2,53 | 2,30 | 1,36 |
| IL12B/IL23B | 9,93 | 281,85 | 48,32 |
| IL23A | 45,28 | 31,19 | 5,84 |
| IFNG | 2,88 | 1,24 | -1,07 |
| **D22** | **LM** | **SA** | **SP** |
| IL12A | 1,85 | 3,29 | -1,03 |
| IL12B/IL23B | 53,91 | 532,51 | 129,44 |
| IL23A | 25,60 | 22,92 | 4,44 |
| IFNG | 3,11 | 6,30 | 2,46 |
| **D23** | **LM** | **SA** | **SP** |
| IL12A | 2,07 | 3,08 | 1,46 |
| IL12B/IL23B | 33,63 | 219,27 | 102,58 |
| IL23A | 39,98 | 15,65 | 4,03 |
| IFNG | 3,52 | 4,24 | 1,31 |
| **D24** | **LM** | **SA** | **SP** |
| IL12A | 1,89 | 1,50 | -1,31 |
| IL12B/IL23B | 12,56 | 1641,21 | 52,56 |
| IL23A | 36,84 | 26,85 | 3,58 |
| IFNG | 36,41 | 18,16 | 3,89 |
|  |  |  |  |
| **AM+/- SD** | **LM** | **SA** | **SP** |
| IL12A | 1,88 +/- 0,46 | 1,74 +/- 1,70 | 0,25 +/- 1,37 |
| IL12B/IL23B | 22,63 +/- 18,66 | 540,99 +/- 573,09 | 71,14 +/- 38,95 |
| IL23A | 31,34+/- 12,88 | 21,01 +/- 8,08 | 4,08 +/- 1,11 |
| IFNG | 9,53 +/- 13,44 | 5,77 +/- 6,68 | 0,64 +/- 2,56 |
