# Supplementary material for: Gram-positive pathogenic bacteria induce a common early response in human monocytes
Source: BMC Microbiol. 2010 Nov 2;10:275. doi: 10.1186/1471-2180-10-275 (PMC2988769; doi:10.1186/1471-2180-10-275)

**Figure S1**. **Correlation of Fold Change**. Relative expression of 14 genes as determined by real time RT-PCR upon infection plotted against their corresponding microarray values. Results are averaged for all 5 donors. Regression coefficient r^2^ = 0,91.


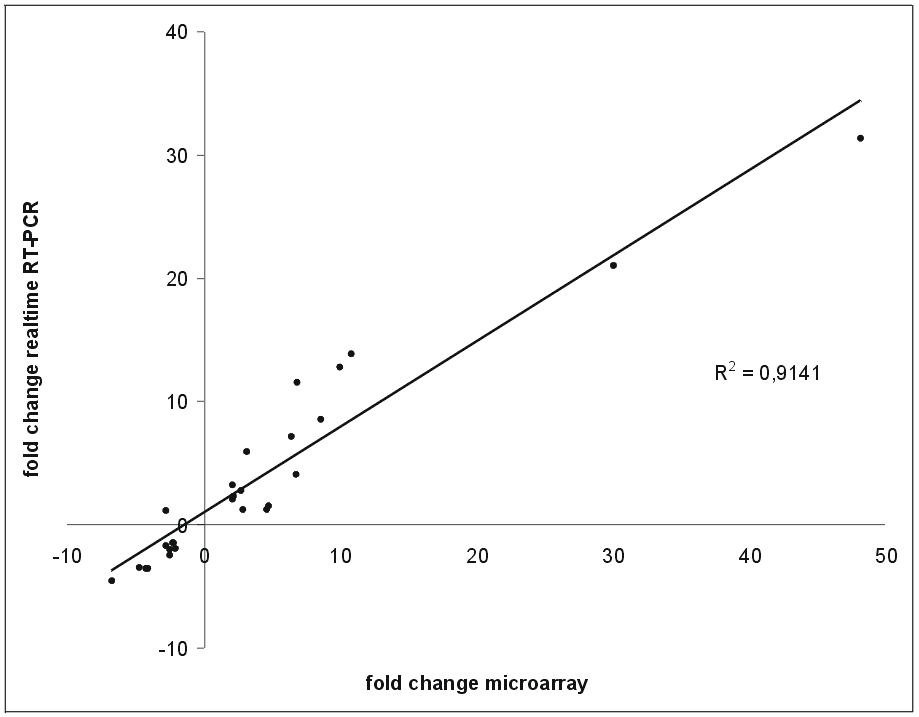

Supplement: Additional file 16 — Table S13. Relative gene expression of IL12A, IL12B/IL23B, IL23A and IFNγ, detected by real time RT-PCR [file 1471-2180-10-275-S16.DOC]
