# Supplementary material for: Gram-positive pathogenic bacteria induce a common early response in human monocytes
Source: BMC Microbiol. 2010 Nov 2;10:275. doi: 10.1186/1471-2180-10-275 (PMC2988769; doi:10.1186/1471-2180-10-275)
Supplement: Additional file 17 — Figure S2. Phenotype of peripheral mononuclear cells before and after CD14+ positive selection. Anti CD11b and anti CD14 antibodies labeling after ficol gradient centrifugation and before and after CD14 positive selection. Percent of positive cells from all viable mononuclear cells. (A) CD11b + : 28% before and 98% positive cells after CD14 + selection. (B) CD14+ : 12% before and 96% positive cells after CD14 + selection [file 1471-2180-10-275-S17.DOC]

**Figure S2.** Phenotype of peripheral mononuclear cells before and after CD14+ positive selection.

Anti CD11b and anti CD14 antibodies labeling after ficol gradient centrifugation and before and after CD14 positive selection. Percent of positive cells from all viable mononuclear cells. (A) CD11b + : 28% before and 98% positive cells after CD14+ selection. (B) CD14+ : 12% before and 96% positive cells after CD14+ selection

CD11b+

CD14+

CD14+ selection

After Ficol

CD14+ selection

After Ficol

A

B
